# A potent myeloid response is rapidly activated in the lungs of premature Rhesus macaques exposed to intra-uterine inflammation

**DOI:** 10.1038/s41385-022-00495-x

**Published:** 2022-03-21

**Authors:** Courtney M. Jackson, Martin Demmert, Shibabrata Mukherjee, Travis Isaacs, Ravyn Thompson, Chase Chastain, Jerilyn Gray, Paranth Senthamaraikannan, Pietro Presicce, Kashish Chetal, Nathan Salomonis, Lisa A. Miller, Alan H. Jobe, Suhas G. Kallapur, William J. Zacharias, Ian P. Lewkowich, Hitesh Deshmukh, Claire A. Chougnet

**Affiliations:** 1 Division of Immunobiology, Cincinnati Children’s Hospital Research Foundation, Department of Pediatrics, University of Cincinnati College of Medicine, Cincinnati, OH USA; 2 Immunology Graduate Program, University of Cincinnati College of Medicine, Cincinnati, OH USA; 3 Department of Pediatrics, Institute for Systemic Inflammation Research, University of Lϋbeck, Lϋbeck, Germany; 4 Division of Neonatology/Pulmonary Biology, The Perinatal Institute, Cincinnati Children’s Hospital Research Foundation, Department of Pediatrics, University of Cincinnati College of Medicine, Cincinnati, OH USA; 5 Divisions of Neonatology and Developmental Biology, David Geffen School of Medicine at the University of California Los Angeles, Los Angeles, CA USA; 6 Division of Biomedical Informatics, Cincinnati Children’s Hospital Research Foundation, Department of Pediatrics, University of Cincinnati College of Medicine, Cincinnati, OH USA; 7 California National Primate Research Center, University of California Davis, Davis, CA USA; 8 Department of Anatomy, Physiology, and Cell Biology, School of Veterinary Medicine, University of California Davis, Davis, CA USA; 9 Division of Pulmonary and Critical Care Medicine, Department of Internal Medicine, University of Cincinnati, Cincinnati, OH USA; 10 Present Address: Immunology Graduate Program, University of Cincinnati College of Medicine, Cincinnati, OH USA

## Abstract

Up to 40% of preterm births are associated with histological chorioamnionitis (HCA), which leads to elevated levels of pro-inflammatory mediators and microbial products in the amniotic fluid, which come in contact with fetal lungs. Yet, fetal pulmonary immune responses to such exposure remain poorly characterized. To address this gap, we used our established HCA model, in which pregnant Rhesus macaques receive intraamniotic (IA) saline or LPS. IA LPS induced a potent and rapid myeloid cell response in fetal lungs, dominated by neutrophils and monocytes/macrophages. Infiltrating and resident myeloid cells exhibited transcriptional profiles consistent with exposure to TLR ligands, as well as cytokines, notably IL-1 and TNFα. Although simultaneous, in vivo blockade of IL-1 and TNFα signaling did not prevent the inflammatory cell recruitment, it blunted the lung overall inflammatory state reducing communication between, and activation of, infiltrating immune cells. Our data indicate that the fetal innate immune system can mount a rapid multi-faceted pulmonary immune response to *in utero* exposure to inflammation. These data provide mechanistic insights into the association between HCA and the postnatal lung morbidities of the premature infant and highlight therapeutic potential of inflammatory blockade in the fetus.

## Introduction

Chorioamnionitis is a histopathologic term indicating inflammation of the chorion, amnion, or both^1^. Up to 25–40% of preterm births are associated with histological chorioamnionitis (HCA), and >90% of very preterm infants (~24 weeks gestation), are exposed to HCA^2,3^. Importantly, HCA is associated with neonatal mortality, sepsis, respiratory disease, and neurodevelopmental problems^4,5^. However, the mechanisms responsible for the link between HCA and poor health outcomes in children is unclear. HCA leads to accumulation of microbial products and elevated production of pro-inflammatory mediators within the amniotic fluid. Due to *in utero* aspiration/swallowing of the amniotic fluid and the mediators it contains, an inflammatory response is triggered in the mucosae of the developing fetus^5,6^. Such fetal mucosal inflammation, has been described in fetal rodents, rabbits, pigs, and sheep, and is marked by elevated levels of pro-inflammatory cytokines, and damage to the lung and intestine^5^. However, the immune ontogeny in these species is distinct from that in humans, and as such, knowledge about the cells and pathways that constitute the fetal inflammatory response in inflammation-exposed human fetuses, remains limited.

In contrast, non-human primates (NHP) are remarkably similar to humans in most biological aspects. We previously showed that intra-amniotic (IA) injection of LPS in pregnant Rhesus macaques leads to placental inflammation that phenocopies severe human chorio^7,8^. Elevated IL-1β and TNFα levels have also been observed in the amniotic fluid and cord blood^9,10^ and levels of inflammatory cytokines in the lung of HCA-exposed Rhesus macaque fetuses have been described in previous studies^8,11,12^. IL-1β and TNFα are thought to be particularly pathogenic as IA administration of either cytokines is sufficient to induce chorio^11,12^ and others have shown IL-1β involvement in multiple aspects of LPS-induced fetal inflammation^7,8,12,13^. Although such responses likely contribute to the increased incidence of postnatal pulmonary and intestinal morbidities associated with HCA (rev. in^4,5^), the cellular dynamics of the fetal mucosal immune responses were not studied in-depth in these studies. In the present study, we used state-of-the-art and complementary technologies to comprehensively describe HCA-induced fetal mucosal immune responses and delineate the individual roles, of IL-1β and TNFα in HCA-induced fetal pathology using the cross-reactive IL-1 receptor antagonist (IL-1RA) and a blocking anti-TNFα Ab given individually or in combination. The results of this study represent a first step towards the development of a therapeutic regimen to counter the known deleterious effects of exposure to fetal inflammation to log term health outcomes in newborns.

## Results

### IA LPS triggers a rapid infiltration of myeloid cells into the fetal lung

**Fig. 1:**
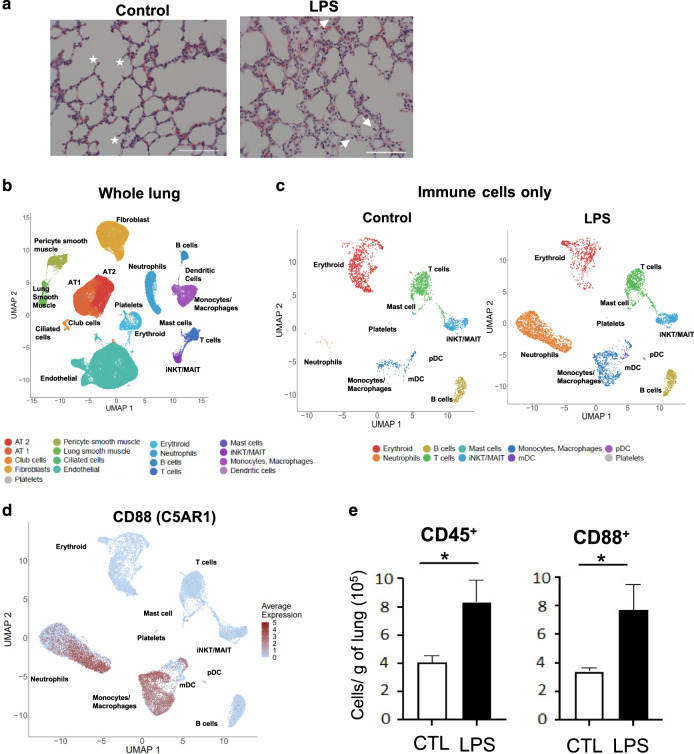
Analysis of the fetal lung 16 h post IA LPS exposure. **a** Representative 20x H&E stain of fetal lung from control and IA LPS-exposed fetuses; stars denote secondary septa, arrows represent infiltrating cells in alveolar space and scale bar is 100 μm. **b–f** The left lobe of fetal lung was processed into single-cell suspensions and submitted for single-cell sequencing. **b** UMAP from combined control and LPS- exposed animals (2 animals/condition). **c** Control (left) and IA LPS (right) exposed animals UMAP of re-clustered immune cell populations. **d** Feature plot of *C5AR1* expression across hematopoietic cell populations. **e** Absolute number of hematopoietic (CD45+) and myeloid (CD88+) cells in control and IA LPS was calculated based on flow cytometric analyses of fetal lungs (*n* = 4). Each symbol represents one animal, with mean (SEM) cells/g tissue displayed; Student’s unpaired *t* test, *p < 0.05.

To model HCA, pregnant Rhesus macaques were given IA LPS or saline at approximately 80% gestation (Supplementary Table S1). Sixteen hours post-IA injections, fetal lungs were collected for analysis (see Supplementary Fig. S1 the schematic representation of lung processing). Compared to lungs from saline-exposed fetuses (hereafter referred to as controls), lungs from IA LPS-exposed fetuses (hereafter referred to as IA LPS), displayed signs of inflammation with cell infiltration into the lung interstitium and increased levels of many pro-inflammatory cytokines, such as TNFα and IL-1β, in the IA-LPS fetal lungs (see Supplementary Tables S2 and S3). Secondary alveoli septa formation was diminished (Fig. 1a). Decreased alveolar space (Fig. 1a) was also observed in IA-LPS animals.

Next, we evaluated the global transcriptional response in the lung in response to IA LPS (see Supplementary Table S4 for demographic data and sequencing metrics) using single-cell RNA sequencing (scRNAseq). Cell clusters identified in the fetal lungs were annotated using signature genes from published scRNAseq atlases^14,15^ (see Supplementary Fig. S1). These analyses revealed distinct immune and non-immune cell populations in both control and IA LPS fetuses (Fig. 1b, Supplementary Table S5). In the lung of IA LPS fetuses there was robust accrual of neutrophils and monocyte/macrophages (Fig. 1c), confirmed by expression of the myeloid-associated *C5AR1* gene, which encodes CD88 (Fig. 1d), compared to controls. This mirrored the flow cytometric analyses of lung cell suspensions wherein the frequency of CD45^+^ hematopoietic cells, particularly CD88^+^ cells (Fig. 1e), was higher in the lungs of IA LPS fetuses than in the controls (see Supplementary Fig. S2 for gating strategy). Frequencies of myeloid dendritic cells (mDCs) and plasmacytoid DCs (pDCs) remain unchanged after IA LPS (Supplementary Fig. S3). However, these DCs demonstrated activated phenotype in IA LPS-exposed fetuses, marked by increased expression of HLA-DR (Supplementary Fig. S3A, B). In contrast, the frequency of B cells or different T cell populations, including regulatory T cells, remained uchanged in the lungs IA LPS fetuses as quantified by flow cytometry and scRNAseq analyses (Supplementary Fig. S4A–D). Additionally, studies of differentially expressed genes (DEGs) uncovered relatively minor changes in these populations in IA LPS fetuses (Supplementary Fig. S4B, D and Supplementary Table S6). Thus, IA LPS rapidly altered fetal innate immune cell populations in the lung, with a rapid increase in myeloid cells but minimal change in the frequencies of lymphoid cells.

### IA LPS promotes recruitment of inflammatory monocytes into the fetal lung and activates the resident macrophages

**Fig. 2:**
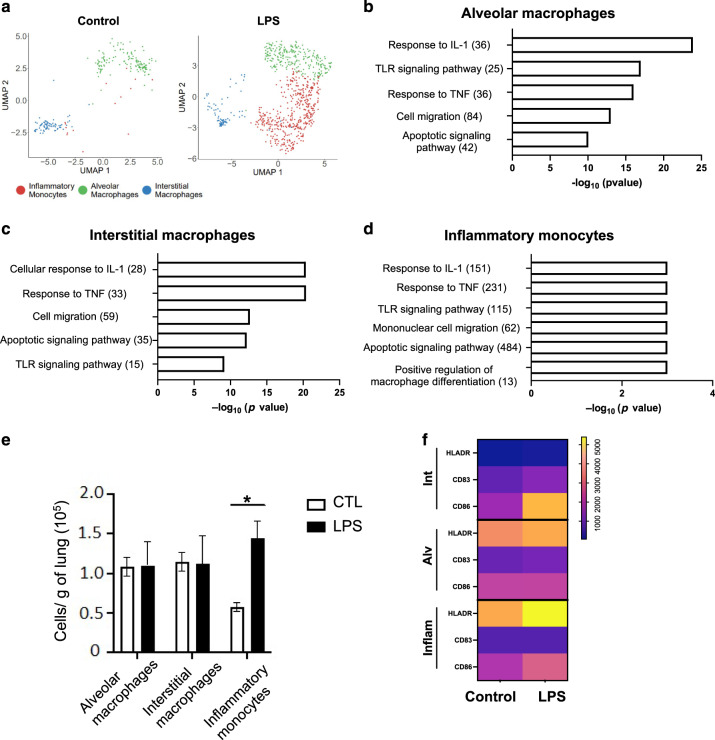
Fetal lung monocyte/macrophage response to IA LPS. **a** UMAPs of fetal lung myeloid cell populations in control (left) and IA LPS (right) fetuses. Functional enrichment analysis of upregulated biological processes based on differentially expressed genes (**b**: alveolar, **c**: interstitial macrophages) using ToppFun. **d** ssGSEA analysis of enriched gene ontology (GO) terms in inflammatory monocytes. Numbers in parentheses represent the total number of genes for each process. **e** Absolute number of macrophages in control and IA LPS was calculated based on flow cytometric analyses of fetal lungs (*n* = 4/group). Mean (SEM) cells/g tissue are shown; Student’s unpaired *t* test, *p < 0.05. **f** Activation markers expression by lung monocyte/macrophage subpopulations was analyzed by flow cytometry (see Supplementary Fig. S5B for representative flow plots). Data are represented as heatmap, the scale representing the range of geometric mean fluorescence.

Based on the expression of published marker genes (Fig. 2b, Supplementary Fig. S5), we identified two distinct macrophage populations in the control lungs, alveolar macrophages and interstitial macrophages (Fig. 2a). In IA LPS lungs we additionally observed a distinct population of monocytes/macrophages with characteristics of inflammatory monocytes (Fig. 2a, b). LPS treatment altered the transcriptomic response in the alveolar and interstitial macrophages (Supplementary Tables S7 and S8), enriching transcripts of genes associated with TLR signaling pathways, and response to cytokines, notably IL-1 and TNF (Fig. 2b, c, Supplementary Tables S9 and S10 for a complete list of genes). In addition, gene transcripts associated with apoptotic signaling and cell migration were also increased in IA-LPS lungs (Fig. 2b, c).

The absence of inflammatory monocyte clusters in the control lungs precluded DEG analysis between the control and IA-LPS lungs for the inflammatory monocytes. However single-sample gene set enrichment analysis (ssGSEA) of inflammatory monocytes in IA-LPS lungs similarly revealed an enrichment of genes related to TLR, IL-1, and TNF signaling, as well as cell migration and apoptosis (Fig. 2d, Supplementary Table S11 for a complete list of genes expressed and enrichment scores). Flow cytometry analyses confirmed the scRNAseq data, demonstrating an increased presence of inflammatory monocytes but similar numbers of alveolar and interstitial macrophages in IA LPS lungs compared to controls (Fig. 2e). All three populations in IA LPS lungs exhibited higher expression of activation markers (Fig. 2f and Supplementary Fig. S5B, for flow cytometry representative examples). Collectively these data demonstrate a robust inflammatory response induced by IA-LPS in fetal lung myeloid cells.

### Recruited neutrophils into the fetal lung have a profile of activation suggesting both direct LPS and indirect cytokine-mediated pathways of activation

**Fig. 3:**
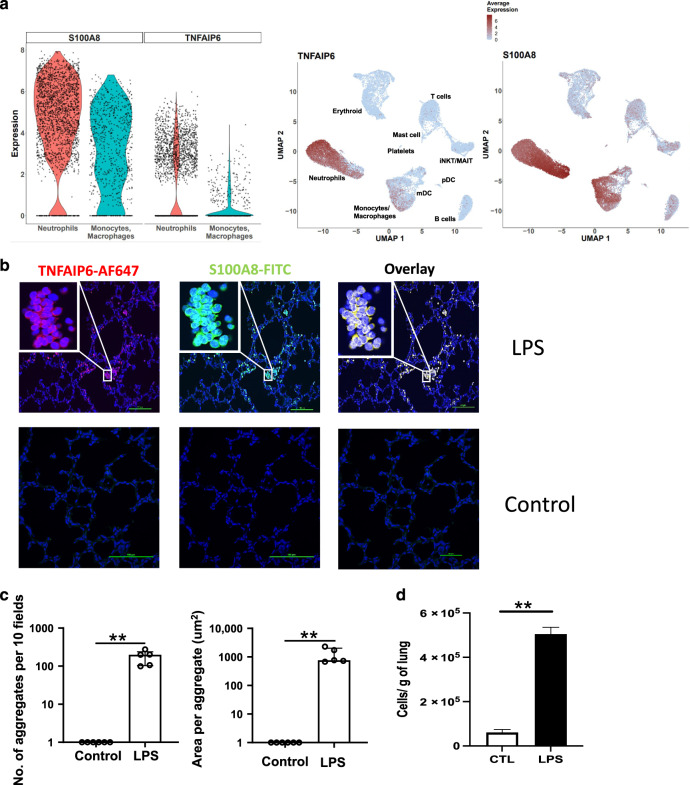
Fetal lung neutrophil response to IA LPS. **a** Violin (left) and feature (right) plot of TNFIAP6 and S100A8 expression in fetal lung neutrophils and monocyte/macrophage population following IA LPS. **b** Immunofluorescence of IA LPS-exposed fetal lung stained for S100A8 and TNFAIP6 (40X, Insert: 100X); scale bar is 100 μm. **c** Neutrophil aggregate count (left) and area (right) in the fetal lungs of control and IA LPS fetuses. Data presented as median and interquartile range, Mann–Whitney *U* test; **p ≤ 0.01. **d** Absolute number of neutrophils in control and IA LPS was calculated based on flow cytometric analyses (*n* = 4) (see Supplementary Fig. S6D for representative flow plot). Data are presented as mean (SEM) cells/g tissue, Student’s unpaired *t* test; **p ≤ 0.01.

Our data also revealed a robust neutrophilic response in the fetal lung after IA LPS. scRNAseq analyses identified *TNFAIP6* (TNF Alpha Induced Protein 6) expression as one of the most specific markers for neutrophils (Fig. 3a, Supplementary Fig. S6A). Although *S100A8* was highly expressed in most neutrophils, it was also expressed by monocyte/macrophages (Fig. 3a). We confirmed by IHC the presence of multiple TNFAIP6^+^S100A8^+^ cells in the IA LPS fetal lungs and not in the control lungs (Fig. 3b). These cells had neutrophilic morphology, forming aggregates within alveolar spaces (Fig. 3b, c). We confirmed these data by staining with CD68 and HLA-DR, as we had found that CD68 is highly expressed in both fetal neutrophils and monocytes/macrophages, with only the latter expressed HLA-DR. A significantly increased number of aggregated CD68^+^HLA-DR^−^ cells, with a neutrophilic morphology, were found in the IA LPS-exposed lungs (Supplementary Fig. S6B, C). The increased absolute number of neutrophils in IA LPS was also confirmed by flow cytometry (Fig. 3d and Supplementary Fig. S6D for flow cytometry representative examples). Furthermore, while neutrophils were largely absent in the alveolar wash (AW) of control fetuses, they were abundant in that of IA LPS fetuses (median 0.0 vs. 1.1 ×10^8^ cells/kg in control (*n* = 18) and IA-LPS (*n* = 13), respectively, *p* < 0.0001, Mann–Whitney test).

**Fig. 4:**
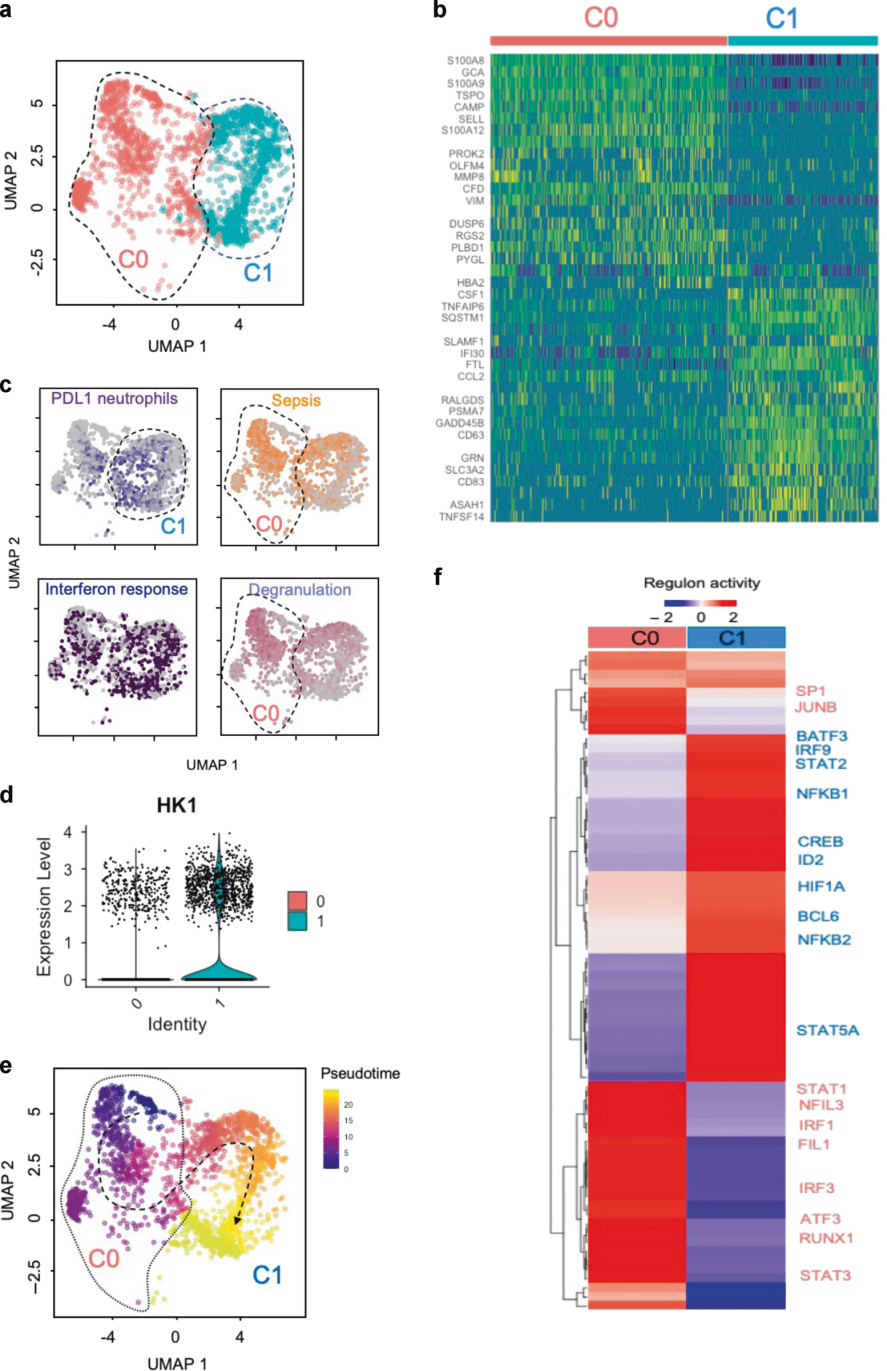
Transcriptomic program of the neutrophils recruited in IA LPS-exposed lungs. **a** UMAP embedding of neutrophils extracted from a larger dataset of lung immune cells colored by clusters. **b** Row-scaled expression of the highest DEGs in each cluster (Bonferroni-adjusted *p* values < 0.05). **c** UMAP embedding of neutrophils colored by average expression of genes associated with exhaustion (PD-L1, purple), sepsis (orange), interferon response (blue) and neutrophil degranulation (lavender). These gene signatures were derived from public gene expression datasets (see Methods). **d** Violin plot of HK1 expression in fetal lung neutrophils following IA LPS. **e** UMAP embedding of neutrophils colored by pseudotime with overlaid trajectory. **f** Row-scaled regulons activity for neutrophil clusters. k-means clustering was used to arrange clusters and regulons.

We next analyzed the transcriptomic program of the recruited neutrophils. Until recently, circulating and tissue neutrophils were considered a homogeneous population with a defined array of functions. However, many studies have challenged this concept revealing significant heterogeneity in this population^16–19^. Our analyses are consistent with the idea of functional heterogeneity, as they show the presence of two distinct neutrophil populations in the lungs after IA LPS (Fig. 4a). To identify these populations, we used module scores^20^, reflecting the average expression of all genes related to neutrophil development, maturation, and activation using published gene signatures from granulocytes during homeostasis and in the setting of sepsis^21^.

Cluster 0 (C0) cells expressed high levels of degranulation genes (*S100A8/9, CAMP, MMP8, OLFM4*, *SELL)*. In contrast, cells in cluster 1 (C1) expressed hallmark markers of an inflammatory response (*IL1A, IL1B, CCL2, CCL20, HIF1a*) and exhaustion (PD-L1) (Fig. 4c, Supplementary Fig. S7A). Cluster 1 cells also exhibited gene transcripts associated with glycolysis, a marker of hyperinflammatory responses^22^ (see Fig. 4d showing a robust expression of *HK1*, which encodes the glycolytic hexokinase-1, in cluster 1). In addition, there was broad induction of genes related to the interferon response, IL1 and TNF activation, and TLR signaling in both clusters (Supplementary Table S12). The developmental relationship among these two clusters was predicted by cellular trajectory analysis^23,24^. Pseudotime analysis supported a continuum of differentiation from mature neutrophils (C0) to senescent, hyperinflammatory neutrophils (C1) (Fig. 4e, Supplementary Fig. S7B). Regulon activity analyses also showed activation of many different pathways in C0 versus C1 neutrophils, further confirming lung neutrophil heterogeneity. Regulatory networks anchored by defense response-associated transcription factors, for example, *NFKB, IRF9, STAT5A, BATF3, and HIF1A*, were enriched in C1 neutrophils (Fig. 4f). In contrast, several regulatory networks, notably those anchored by SPI1 (encoding PU.1), ATF3, or RUNX1 shown to protect against exuberant innate immune responses^25–27^, were active in C0 neutrophils.

**Fig. 5:**
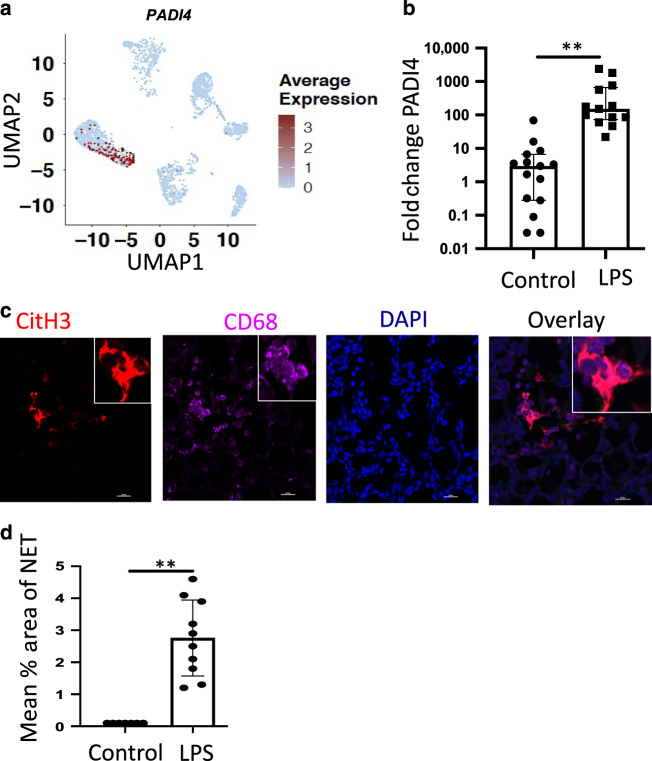
NET formation in LPS-exposed lungs. **a** Feature plot of *PADI4* expression across hematopoietic cell populations of IA LPS-exposed fetuses. **b** *PADI4* mRNA expression was analyzed by RT-PCR in the fetal lung of control animals compared to IA-LPS injection. **c** Representative immunofluorescence staining of NETs in IA-LPS-exposed fetal lung (60X, Insert: 100X); scale bar is 50 μm. **d** Average area of NET in the control and IA LPS fetal lungs. Each symbol represents one animal, with median and interquartile ranges displayed, Mann–Whitney *U* test; **p ≤ 0.01.

Neutrophils form extracellular traps (NETs) in response to bacteria or bacterial products. While NETosis is an essential component of neutrophil antimicrobial activity, it contributes to tissue injury, particularly in the context of sterile inflammation (rev. in^28,29^). Cord blood neutrophils from preterm and term infants are deficient in NET formation in response to ex vivo stimuli (rev. in^30^). Nevertheless, the ability of fetal neutrophils to form NETs in vivo is unclear. Consistent with the essential role of peptidyl arginine deiminase 4 (PAD4) in NETosis^31,32^, we found that *PADI4* was uniquely expressed in neutrophils in the lungs of IA LPS fetuses (Fig. 5a), and its upregulation was confirmed by quantitative RT-PCR in a larger number of animals (Fig. 5b). As NETosis is characterized by the extrusion of chromatin material loaded with citrullinated histones and granule contents, we quantified the expression of extracellular citrullinated histone 3 (H3cit), in the lungs of IA LPS and control animals by IHC. As expected, due to the low number of neutrophils, H3cit was almost undetectable in control lungs. In contrast, long fibrous projections expressing H3cit were detectable in the lungs of IA LPS fetuses (Fig. 5c). These projections were present in all IA LPS animals, and the mean NET area was significantly larger in LPS animals compared to controls (Fig. 5d). Confirming the essential role of PAD4 in NETosis, *PADI4* mRNA expression was significantly correlated with the NET area (*r* = 0.84, *p* = 0.044, Spearman correlation test).

### IL-1 and TNFα signaling partially drive fetal lung inflammation

**Fig. 6:**
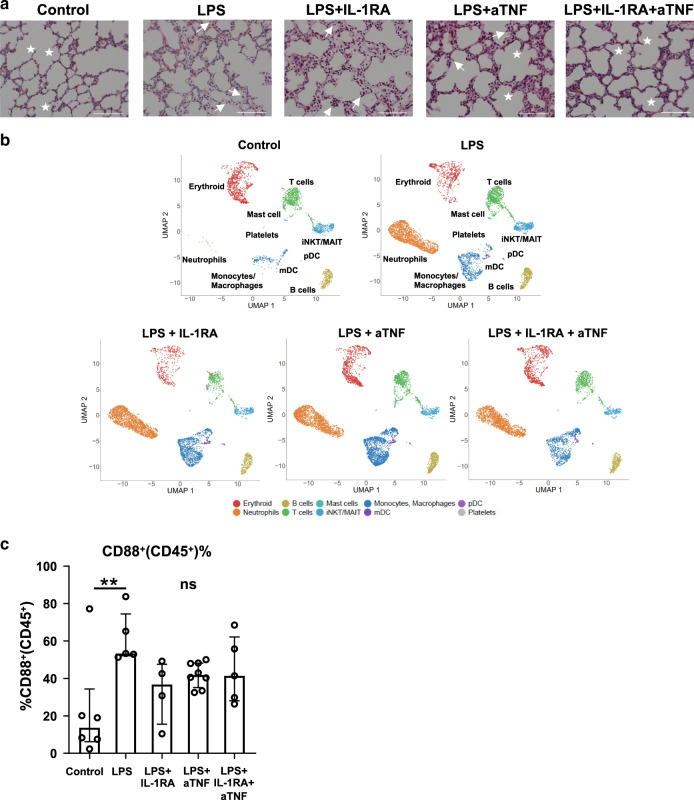
Histologic changes but no change in myeloid cell recruitment in fetal lung following blocking IL-1 and TNF signaling. **a** Representative 20x H&E sections of lungs from controls, LPS, LPS + IL-1RA, LPS + anti-TNF, and LPS + IL-1RA + anti-TNF exposed animals; stars denote secondary septa, arrows represent cells in alveolar space and scale bar is 100 μm. **b** UMAPs of the lung hematopoietic cells across treatments (*n* = 2-3/condition). **c** Percentage of myeloid (CD88^+^) cells within lung CD45^+^ cells. Each dot represents one animal. Median and interquartile ranges are displayed, Kruskai–Wallis test; **p ≤ 0.01. NS not significant.

IL-1 and TNFα are critical in the pathogenesis of chorio^8,11,12,33,34^. Our scRNAseq analyses revealed gene signatures consistent with IL-1 and TNFα signaling in both the lung monocyte/macrophages and neutrophils of IA LPS animals. We, therefore, blocked IL-1 signaling with IL-1RA and TNFα with the anti-TNF Ab Adalimumab, administered via subcutaneous and IA route to the pregnant dams. We also tested their combined effect (see Supplementary Fig. S8A for experimental scheme). As shown in Supplementary Fig. S8B, and consistent with previously published data, IL-1RA and anti-TNF, alone or in combination, blunted chorioamnionitis^8,33,35^. These blockades also significantly decreased mRNA expression of IL-1β and TNF-α in fetal membranes (Supplementary Fig. S9). In contrast, cytokine abundance in the AW and lung cytokine mRNA expression were only partially diminished with any of the treatments (Supplementary Tables S2 and S3). IL-1RA or anti-TNF inhibitors alone did not diminish LPS-induced cell infiltration and lung interstitium thickening, but their combination decreased lung interstitium thickening (Fig. 6a). Overall infiltration of myeloid cells into the lung was not blunted by any treatment, as shown by scRNAseq and flow cytometry analyses (Fig. 6b, c).

**Fig. 7:**
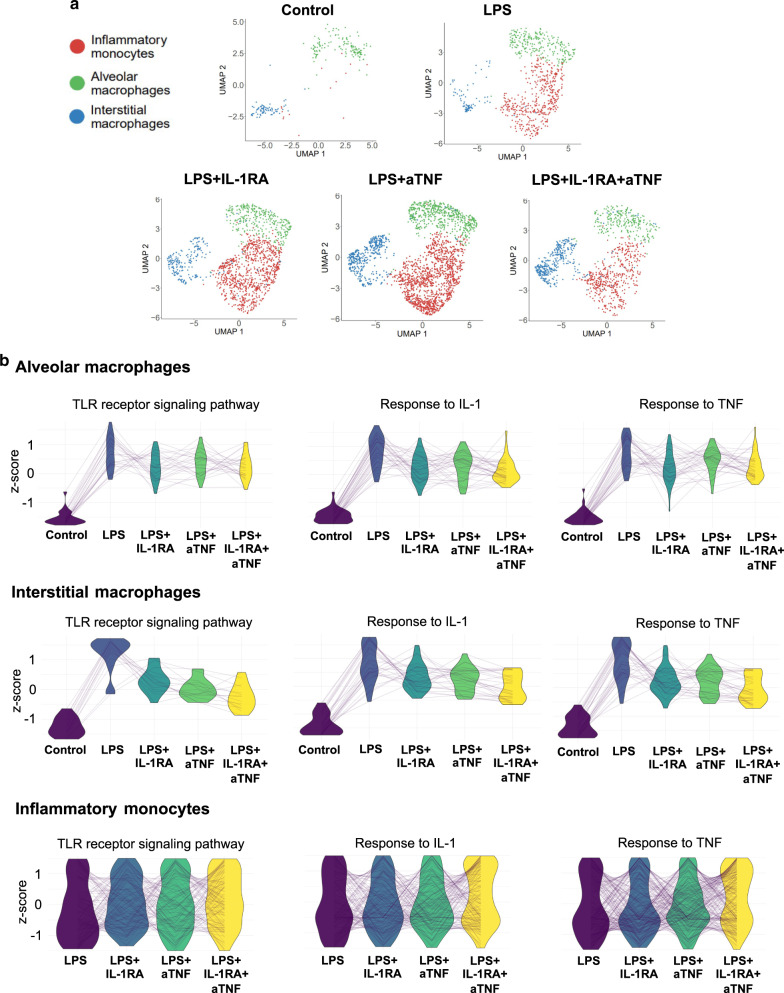
Fetal lung monocyte/macrophage population across treatment conditions. **a** UMAPs of fetal lung monocyte/macrophage populations across treatment conditions. **b** Parallel coordinate plots of scaled expression of representative genes in select biological processes across treatment conditions in alveolar, interstitial macrophages and inflammatory monocytes.

Although the frequency of monocyte/macrophage populations remained unchanged in the lung of fetuses treated with IL-1RA or anti-TNF inhibitors, compared to IA LPS fetuses (Fig. 7a), the blockades, above all by the combined blockade, diminished some of the biological processes activated by IA LPS (Fig. 7b, Supplementary Fig. S10A–C). Notably, the expression of genes associated with the TLR, IL-1, and TNFα signaling pathways decreased in the alveolar and interstitial macrophages, particularly in the fetuses that had received the combined treatment (Fig. 7b). Blockades also reduced the expression of genes associated with cell migration and apoptotic signaling in these cells (Supplementary Fig. S10A, B). However, the blockades did not blunt these processes in the inflammatory monocytes (Fig. 7b, Supplementary Fig. S10C). Treatment with IL-RA, alone or in combination, also reduced activation of the mDC population (Supplementary Fig. S11).

**Fig. 8:**
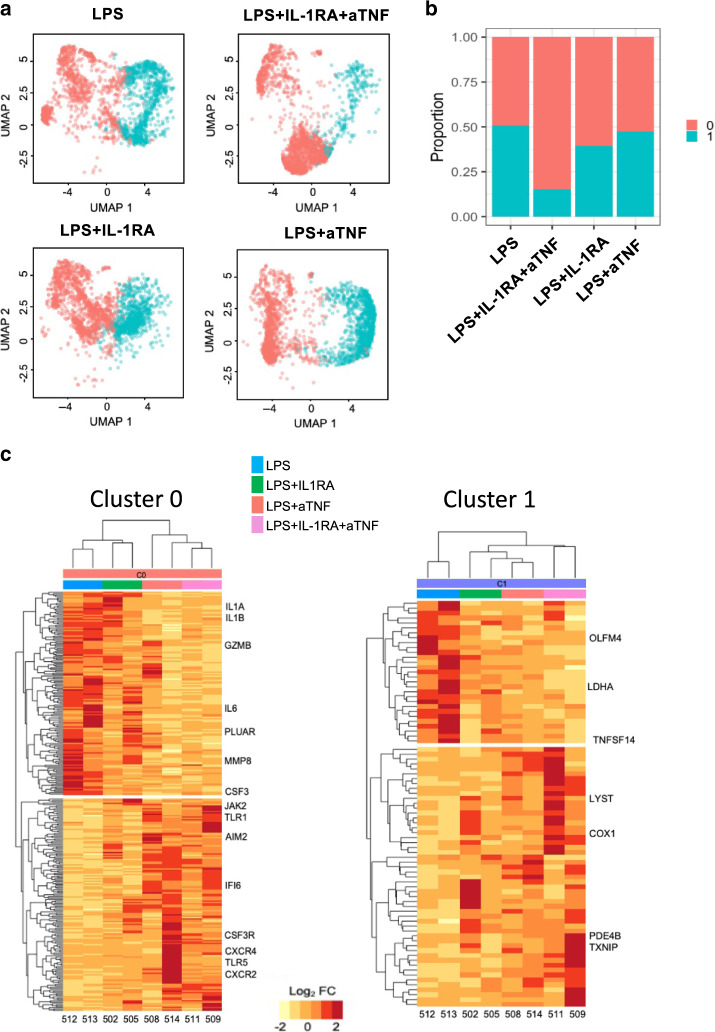
Fetal neutrophils across treatment conditions. **a** UMAPs of fetal lung neutrophils across treatments (*n* = 2–3/condition). **b** Proportions of each neutrophil cluster in the different conditions. **c** Row-scaled expression of DEGs in the pulmonary neutrophils (cluster 0 or cluster 1) across treatments, normalized against all subjects. k-means clustering was used to arrange subjects and transcripts (Benjamini and Hochberg-adjusted *p* values < 0.01, log2 fold change > 2, Wald’s test).

Overall, neutrophil migration into the lung was not diminished by any of the blockades (Fig. 8a). IL1RA or anti-TNF alone had only a minimal impact on the neutrophil transcriptomic profile (Fig. 8a, b). However, the combined blockade decreased the abundance of senescent, hyperinflammatory neutrophils (C1 neutrophils) (see Fig. 8a, b). Transcripts of genes related to leukocyte apoptosis, such as *CTSC*, *PYCARD*, and *C1QBP*, respiratory burst, such as *MPO*, *NCF1*, and *NCF2*, and cellular stress and ubiquitination, such as *HIF1A*, *EGR1* and *TANK*, which were increased in neutrophils of LPS treated macaques, were decreased after combined blockade (Fig. 8c, Supplementary Tables S13 and 14). Expression of *PDI4* mRNA and NETosis was not affected by the combined blockade (Supplementary Fig. S12).

### Extensive communication between monocytes/macrophages and neutrophils defines the LPS signaling milieu and is suppressed by combined blockade of IL-1 and TNF signaling

**Fig. 9:**
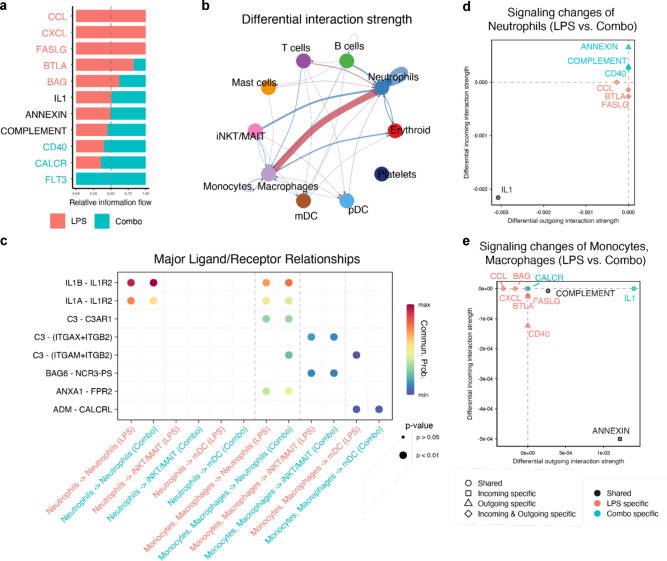
Signaling relationships among immune cells during LPS-induced lung injury compared to combined TNFα and IL-1R blockade. **a** Major signaling pathways changed between LPS and combination (+IL-1RA and anti-TNF) blockade (Combo). **b** Differential interactions strength of cell signaling relationships between immune cells. Signals increased in LPS are shown in red, those increased in combination blockade in blue. **c** Major signaling ligands involved in monocytes/macrophages and neutrophils comparing LPS and combination blockade. Comparison of major incoming and outgoing signals in neutrophils (**d**) and monocytes/macrophages (**e**) during LPS or LPS+ combination blockade. All analyses were performed with CellChat (see Methods).

To understand the impact of LPS on immune cell interactions in the lung, we utilized the ligand-receptor signaling package CellChat^36^. Comparison of immune cell interactions showed that LPS-induced extensive TNF, FASL, and CCL/CXCL signaling in pulmonary immune cells (Fig. 9a), primarily due to interactions between macrophage/monocytes and neutrophils (Fig. 9b), consistent with the vital role for these cells in the LPS response. Closer evaluation of these interactions shows that IL-1 signaling is the most significant pathway involved in neutrophil-neutrophil and monocyte-neutrophil interactions (Fig. 9c). In animals treated by both inhibitors, many of these signals were reduced, concordant with the blunted inflammatory activation seen in the lung. Notably, monocyte/neutrophil signaling was dramatically decreased (Fig. 9b, d, e), despite relatively small changes in cell number in the lungs (Fig. 6c). Interestingly, in the signaling model, IL1 signaling showed no significant net difference between conditions, as the expression of IL1 receptors increased to compensate for the reduction in IL1β expression (Supplementary Tables S2 and S3) and the presence of IL1RA. These findings demonstrate that blocking IL1 and TNF signaling hinders signaling loops between neutrophils and monocytes, which potentiate inflammatory signals.

## Discussion

HCA-exposed premature infants are at increased risk of developing pulmonary morbidities after birth (rev. in^5,37^). Herein, we applied several complementary techniques to comprehensively analyze the fetal lung immune response to HCA in the highly relevant Rhesus macaque model. In this model, shortly after IA LPS, robust inflammation developed in the fetal lungs. Myeloid cells were mainly involved, as the most significant cellular change occurring in the lungs of IA LPS-exposed fetuses was the massive accrual of neutrophils and inflammatory monocytes, cell types absent in the control lungs. In addition, resident myeloid cells, notably the alveolar and interstitial macrophages, exhibited signs of activation, and a shared inflammatory transcriptional program.

Overall, these data indicate that the fetal innate immune system can rapidly mount a strong response, a concept that remains controversial. Indeed, many studies describe functional impairment in fetal/neonatal neutrophils and monocytes/macrophages compared to their juvenile and adult counterparts (rev. in^30,38,39^). Described defects notably include the decreased recruitment of neutrophils and inflammatory monocytes into the lung in response to *E. coli* instillation during the same period, compared to juvenile mice^40^. In contrast, our data in an NHP model argue that fetal neutrophils and inflammatory monocytes can quickly migrate into the pulmonary environment. Neutrophils appeared to localize in the lung in the form of aggregates, a pattern reminiscent of that described in murine models of acute lung injury^41,42^. Similarly, other groups described rapid neutrophil recruitment in fetal sheep or rhesus macaque lungs in response to IA LPS or IL-1β^12,43^. Furthermore, we report that, contrary to what has been described before^30^, fetal neutrophils could form NETs. It is to be noted that the concept of defective functionality of innate immune cells in human neonates is mainly based on limited in vitro studies of stimulated cord blood cells. Likely, these assays did not fully reveal their functional potential, which our in vivo studies illuminated. Another caveat when interpreting data about preterm babies’ immune cell functions is that many of these infants had received steroids, which affect cell behavior and function. We did not administer steroids in our studies, which may have allowed us to observe such robust immune responses.

We evaluated the role of IL-1 or TNFα in fetal mucosal responses to IA LPS. Our data show that blocking either or both of these pathways had only a partial effect on lung inflammation. This was not completely unexpected, as transcriptomic analyses revealed gene signatures consistent with the activation of both LPS- and cytokine-mediated pathways. The partial amelioration of disease by IL-1RA was similar to other HCA models^8,33,34,43^. At the same time, this was the first attempt at evaluating the effect of anti-TNF alone or in combination with IL-1RA.

Combined IL-1β/TNFα inhibition did not block the recruitment of inflammatory myeloid cells into the lung. Several scenarios may explain this data. Other mediators, for example, CCL2, which regulates many aspects of monocyte biology^44^, remain elevated in the lung tissue and AW of treated animals. High levels of IL-8 persisting in the lung and AW despite the blockades may also be responsible for continued myeloid cell recruitment. Neutrophilic chemoattractant molecules, including chemotactic lipids and formyl peptides, complement anaphylatoxins, which are released during inflammation (rev. in^45^) could also play a role. We did not quantify these mediators in fetal lungs or AW. However, we speculate that they were likely released despite the blockades because they are regulated through TLR and cytokine-mediated mechanisms. In contrast to the lungs, myeloid cell recruitment in the placenta was significantly reduced by either IL-1RA or anti-TNF, or the combined blockade^33,35^ (and Supplementary Figs. S8 and 9), suggesting tissue-specific mechanisms controlling cell migration. Alternatively, and not exclusively, the fact that cells recruited in the placenta have a maternal origin, in contrast to fetal cells migrating to the lungs, may also have driven some of these differences.

**Fig. 10:**
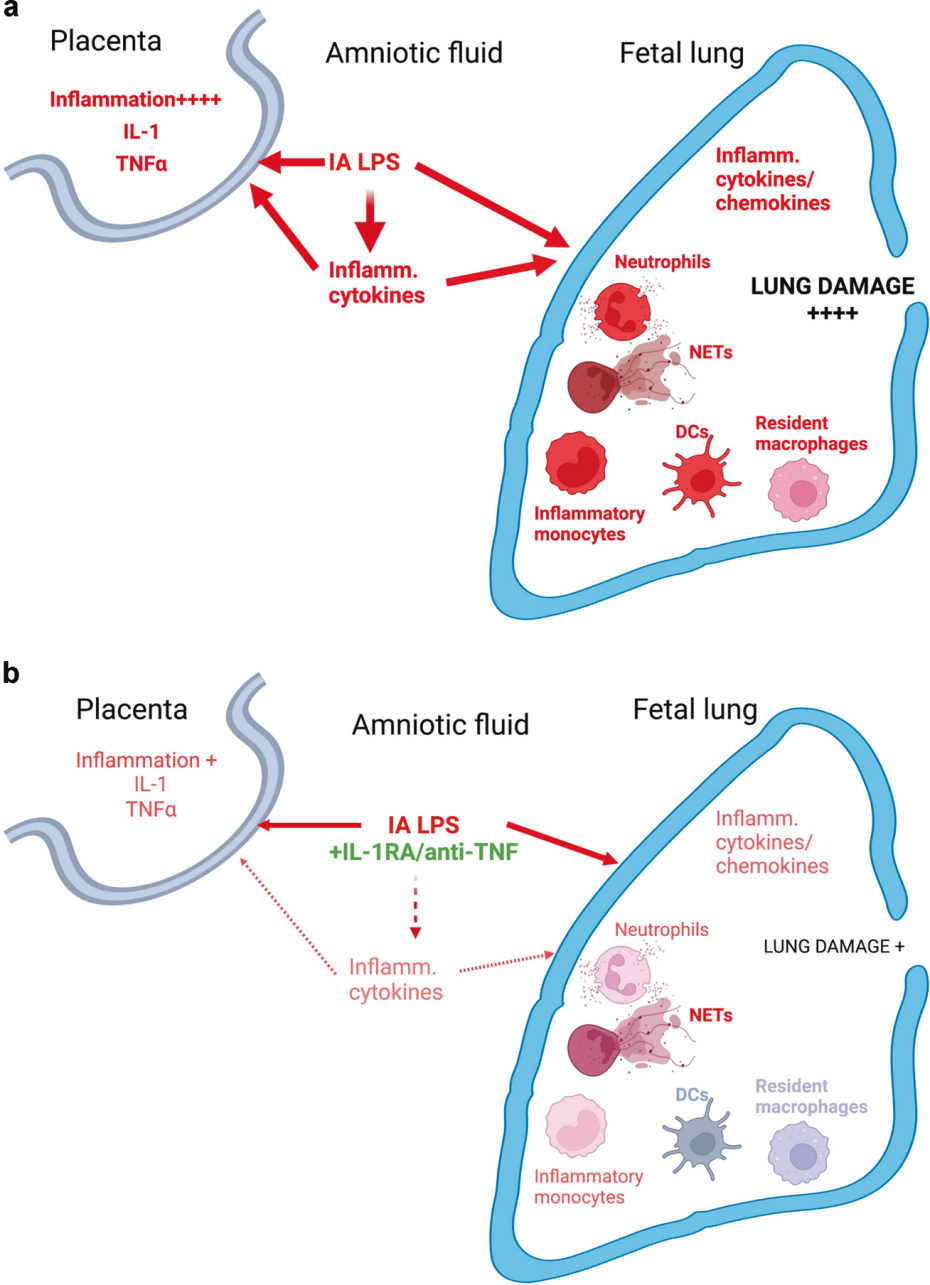
Model of factors contributing to HCA-induced placental and fetal lung inflammation. **a** IA LPS injection induces a mixture of TLR-dependent, and IL-1/TNFα signaling pathway-mediated activation in the placenta (described in more detail in^33,35^). In the lung, IA LPS induces production of inflammatory cytokines/chemokines, recruitment of activated neutrophils and monocytes, as well as activation of resident myeloid cells (macrophages and DCs). Together, these changes contribute to the extensive lung damage observed in HCA-exposed fetuses (described in more detail in^46^). **b** Co-administration of IL-1RA and anti TNFα in the context of LPS exposure blunts placental inflammation. Although inflammatory cell recruitment and NET formation is not significantly diminished, production of inflammatory cytokines/chemokines, activation of recruited and resident myeloid cells is blunted. Lung damage is also significantly decreased by co-administration of IL-1 and TNFα blocking agents. Created with *BioRender.com*.

Nevertheless, the combined blockade remodeled the transcriptomic profile of lung myeloid cells. The expression of gene transcripts associated with the TLR, IL-1, and TNFα signaling pathways in the alveolar and interstitial macrophages and the frequency of hyperinflammatory neutrophils were diminished by the combined blockade. In addition, signaling loops between neutrophils and monocytes were also blocked. Together, these data suggest an overall lower inflammatory state in the lung despite small changes in inflammatory cell numbers. We found that this combined blockade also reduced inflammatory activation of structural lung cells and restored critical signaling networks involved in alveologenesis^46^. These data, taken together, argue that HCA-mediated lung injury results from both TLR-dependent and IL-1/TNFa signaling-mediated pathways (see overall model in Fig. 10).

Overall, our work in a model that phenocopies the main features of human HCA gives novel insights into the inflammation rapidly developing in the fetal lung following such exposure. Our data also emphasize that the fetal lung is remarkably responsive, as it is in close contact with the amniotic fluid, whereas we found that the fetal gut is less affected^47^. This study thus provides more insights into why the most solid associations found between HCA exposure and postnatal morbidities in humans involved mainly the lung (bronchopulmonary dysplasia, wheeziness, and asthma). However, our data also show that the multi-parametric mucosal inflammation that develops in HCA-exposed fetuses is not easily controlled by blocking only one or two inflammatory mediators, emphasizing the need for additional mechanistic studies to understand the dynamic and complex responses that HCA triggers in exposed fetuses.

## Methods

### Animals

The Institutional Animal Care and Use Committee at the University of California, Davis, approved all animal procedures. Normally cycling, adult female Rhesus macaques (*Macaca mulatta*) that already had a normal pregnancy were time mated. Pregnancy, and its maintenance without complication, was confirmed by ultra-sound and examinations carried at ~GD50, GD80 and before experiments were started. Rare twin pregnancies were excluded. At 132 ± 3 day gestation (about 80% of term gestation), pregnant dams received either saline or 1 mg of LPS (Sigma, St. Louis MO) by ultrasound-guided IA injection. IA administration of LPS or saline was performed in mothers of similar weights and ages with fetuses with similar fetal genders and gestational ages (Supplementary Table S1). Fetuses were surgically delivered 16 h later by cesarean section. Delivered fetuses were euthanized with pentobarbital and tissues collected. There were no spontaneous deaths or preterm labor in the animals. Some pregnant dams were given, in addition to IA LPS, either IL-1RA (Kineret® Sobi, Stockholm, Sweden) or Adalimubab (HUMIRA® AbbVie Chicago, IL), or both, administered subcutaneously and IA before IA LPS (see Supplementary Fig. S8).

### Histological evaluation of fetal lung

Inflated lungs (30 cm water pressure) were fixed in 10% formalin immediately following removal. After a series of alcohol and xylene washes, tissues were blocked in paraffin. Paraffin sections were stained with hematoxylin & eosin (H&E) and imaged with a Nikon 90i Upright Widefield Microscope (Nikon Instruments Inc., Melville, NY). 100 µm scale bar added using Nikon Elements Software.

### Immunofluorescence analysis of neutrophils

Upper left lung lobe sections (5 µm thick) were blocked with 4% goat serum in PBS and then incubated 1 h at RT with the primary mouse anti-human Ab diluted in 4% goat serum in PBS. Cross-reactivity of all Ab with Rhesus macaque cells was verified on adult tissues prior to their utilization to stain fetal cells. Abs were titrated for optimal detection of positive populations and MFI. Sections stained with unlabeled Ab were stained with a corresponding secondary goat-anti-mouse Ab conjugated *(*Life Technologies, dilution 1:400) for 1 h. As a negative control, tissues were also stained with the secondary Ab only. Sections were washed and incubated with a Vecta shield autofluorescence reduction kit and mounted with mounting media containing DAPI (Vector Laboratories). Images were acquired using an inverted microscope outfitted with a confocal scan-head (Ti-E and A1R; Nikon). Analyses involving deconvolution and background reduction were done with Nikon Elements Software. Since neutrophils in the sections were mostly in clumps/aggregates, we evaluated the number of neutrophil aggregates in 10 fields per animal. The neutrophils were identified as S100A8-FITC (CF-145) and TNFAIP6-AF594 (polyclonal) (both ThermoFisher Waltham, MA) double-positive cells. The average area covered by neutrophils clumps were also determined and normalized to the total DAPI area of the image per field. CD68-FITC (KP1) (Alligent Dako, Santa Clara, CA) and HLA-DR-AF594 (L243) (BioLegend, San Diego, CA) were also used to quantify neutrophils. NETs were identified using citrullinated histone (CitH3, Abcam, Cambridge, UK).

### Alveolar wash analyses

Alveolar wash was collected from the left lung. Cell-free supernatants was frozen until analyses using the NHP multiplex kit (Millipore Austin, TX). Cell pellets were cytospun and slides were stained with Diff quick. Five fields/slides (100 cells/field) were counted twice.

### Quantitative RT-PCR analyses of mucosal tissues

From frozen lung, total RNA was extracted after homogenizing in TRIzol (Invitrogen, Carlsbad, CA). 1 µg of RNA was used to generate cDNA using the Verso cDNA synthesis kit (Thermo Fisher Scientific). Quantitative RT-PCR was done with a StepOnePlus real-time PCR system and rhesus-specific TaqMan gene expression primers (Life Technologies). Eukaryotic 18S rRNA was the endogenous control used for normalization of the target RNAs. The mRNA fold change was calculated relative to the average value of the control group.

### Fetal lung cell isolation

Left lobe was processed into single-cell suspensions as described^48^. The large airways were removed, and ~500 mg of airway-free tissue was processed in a gentleMACS octo dissociator (Miltenyi Biotec, Auburn, CA), in presence of 5 mL digest buffer. The cell suspension was passed through a 100 µm filter, washed with PBS, red blood cell were lysed and filtered again through a 40 um filter after neutralization.

### Single-cell sequencing of fetal lung cells

Approximately 50,000 lung cells from a subset of animals (Supplementary Table S4) were submitted for single-cell sequencing. Approximately 16,000 cells were loaded into one channel of the Chromium system using the 3 prime v3 single-cell reagent kit (10X Genomics, Pleasanton, CA). Following capture and lysis, cDNA was synthesized and amplified (10X Genomics). The amplified cDNA was used to construct Illumina sequencing libraries that were each sequenced using an Illumina HiSeq 4000 machine. Raw sequencing data was processed aligned to the Rhesus macaque reference Mmul_8.0.1 (Ensembl version 91) with Cell Ranger 3.0.2 (10X Genomics) generating expression count matrix files. After doublet removal with DoubletFinder (version 2.0), R (version 3.6.3) and the Seurat package (version 3.1.0, https://satijalab.org/seurat/), were used to identify common cell types across different experimental conditions. Cells with 25% or more mitochondrial transcripts were removed, as well as cells expressing fewer than 150 or more than 5,000 features. After log-normalization, 5,000 highly variable features were identified per sample using the vst method. Integration was performed using the Seurat FindIntegrationAnchors and IntegrateData functions with default parameter values. The integrated data was scaled, including regressing out mitochondrial percentage and cell cycle variables, followed by application of PCA reduction. A UMAP projection was estimated based on the PCA reduction and the correlation metric. Clustering of cells was done using Seurat FindNeighbors (based on first 30 P.C.s) and FindClusters (resolution = 4). This high-resolution was initially used to define highly discrete immune populations, with the final populations subsequently aggregated into broader, higher confidence cell-types based common marker genes expressed and corresponding UMAP embeddings. To annotate these cell-populations, we used established human and mouse cell-population marker genes annotated by LungMAP, from the AltAnalyze and ToppCell databases, followed by label transfer with cellHarmony against described reference human lung datasets^14,15^. Clusters were orthogonally validated using ICGS2 in AltAnalyze, which does not require a resolution setting, to confirm their presence. For further analyses, Y-chromosomal genes were removed because IA LPS-exposed fetuses were male, whereas animals of both sexes were present in the other experimental groups. Additionally, we checked for the expression of genes *XIST* and *TSIX*, which did not appear in our dataset and seem to not be drivers of differences observed. All identified cell populations were required to have unique reported marker genes to ensure they were not artifacts of “over-clustering”. Differential gene expression analysis was performed in Seurat. Plots were generated using the ggplot2 package (version 3.3.2). DEGs between control and LPS in the monocyte/macrophage cluster with a fold change (FC) of ≥1.2 and adjusted *p* value of ≤0.05 were used for functional enrichment analysis of biological processes and pathways using the ToppFun web portal^49^. Terms with higher expression in LPS are represented as positive log *p* values. Scaled expression of genes for upregulated biological processes terms in the monocyte/macrophage cluster were used to plot parallel coordinate plots using the ggplot2 package.

We also performed single-sample gene set enrichment analysis (ssGSEA) in R using the ssgsea2.0 package (github.com/broadinstitute/ssGSEA2.0) in order to identify enriched gene ontology (GO) gene sets (MSigDB v7.0). Expression values of overlapping genes of the most significant and relevant gene sets were plotted as parallel coordinate plots using ggplot2. Analysis of ligand-receptor relationships was performed using CellChat (version 1.13) with default parameters with the exception of using the [population.size = TRUE] modifier when running the computeCommunProb function^36^. This modification allows use of cell number in calculating communication networks which improves predictions for populations with low or uneven cell numbers between different conditions. Single-cell trajectory analysis was performed with Monocle3 (version 0.2.3.0). Spatial autocorrelation analysis as implemented in Monocle3 was used to determine genes that most strongly vary along the pseudo-time trajectory. The scRNA-seq data have been deposited in the Gene Expression Omnibus (GSE169390, reviewer token:qxatqgkyhxyrhkl).

### Flow cytometry

A cocktail of conjugated Ab was used to phenotype lung cell suspensions for multi-parameter flow-cytometry (see Supplementary Table S5). Lung cell suspensions were treated with human IgG to block Fc-receptors and stained for surface markers. Cells were then washed and treated with fixation/permeabilization buffer (ThermoFisher). Following permeabilization, cells were stained for intracellular markers, washed, and resuspended in PBS. Cells were collected on a FACS Fortessa and analyzed with BD FACSDiva Software v8.0.1 (BD Biosciences).

### Statistical analyses

Prism 8 (GraphPad, La Jolla, CA) was used to graph and analyze data for statistical significance. Data were first checked for normality and values expressed as either mean ± SEM or median and interquartile range. Statistical differences between 2 groups were analyzed using Mann–Whitney *U*-tests or Student’s *t* test and two-tailed tests were used. For comparison of more than two groups, Kruskal–Wallis or One-way ANOVA were used. Results were considered significantly different for *p* values ≤ 0.05. In some experiments, due to the limited number of samples per group, we also report trends (*p* values between 0.05 and 0.1).

## Supplementary information

Supplementary Tables

Supplementary Information
