# Supplementary material for: A potent myeloid response is rapidly activated in the lungs of premature Rhesus macaques exposed to intra-uterine inflammation
Source: Mucosal Immunol. 2022 Mar 21;15(4):730–44. doi: 10.1038/s41385-022-00495-x (PMC9259482; doi:10.1038/s41385-022-00495-x)
Supplement: Supplementary file 2 — Supplementary Information [file 41385_2022_495_MOESM2_ESM.pdf]

Fig. S1

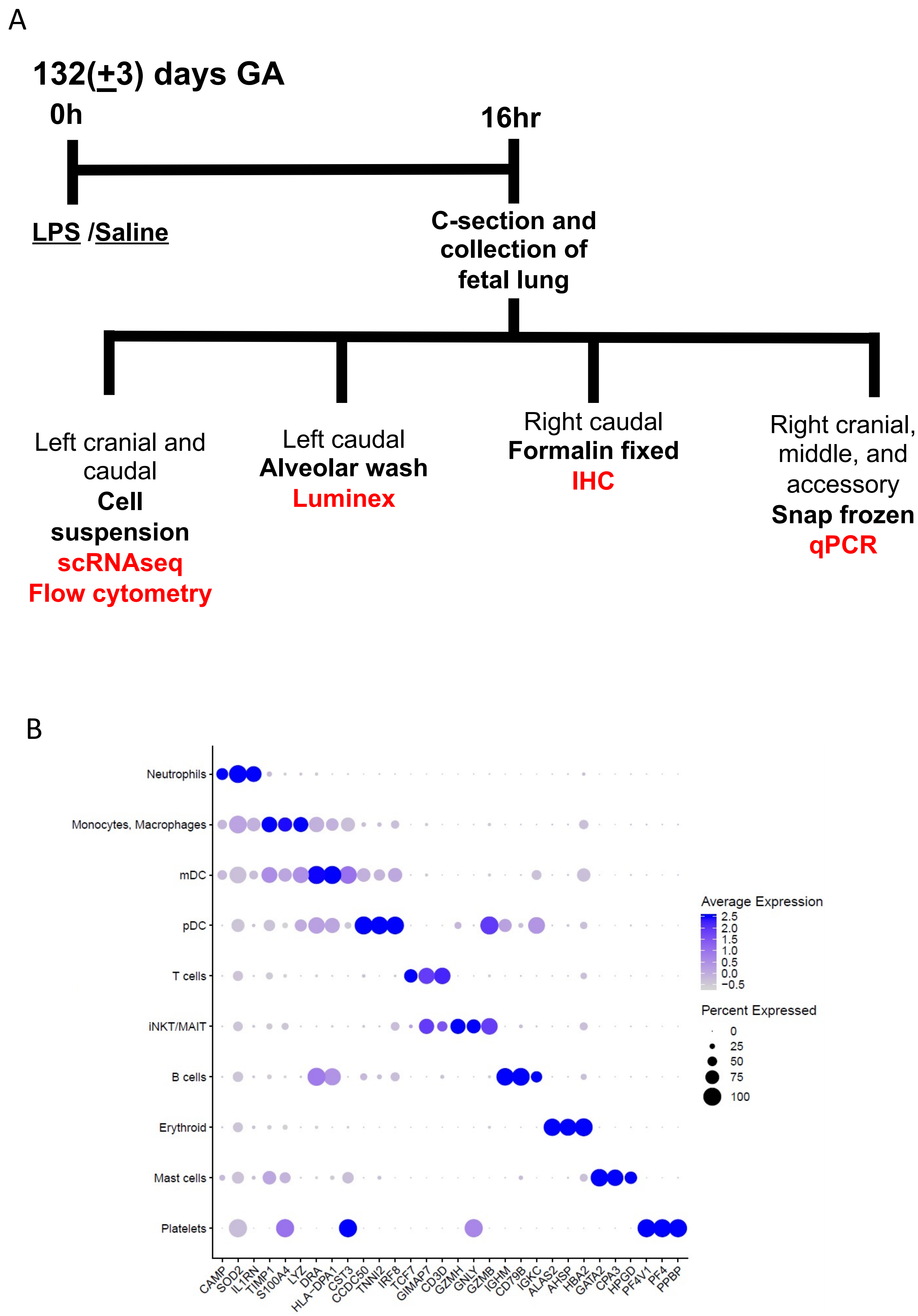

Fig. S1: **(A)** Schematic representation of fetal lung analyses. Processing method (in bold) and applications (in red) are indicated. **(B)** Canonical cell type markers used in the identification of hematopoietic populations in scRNAseq experiments.

Fig. S2

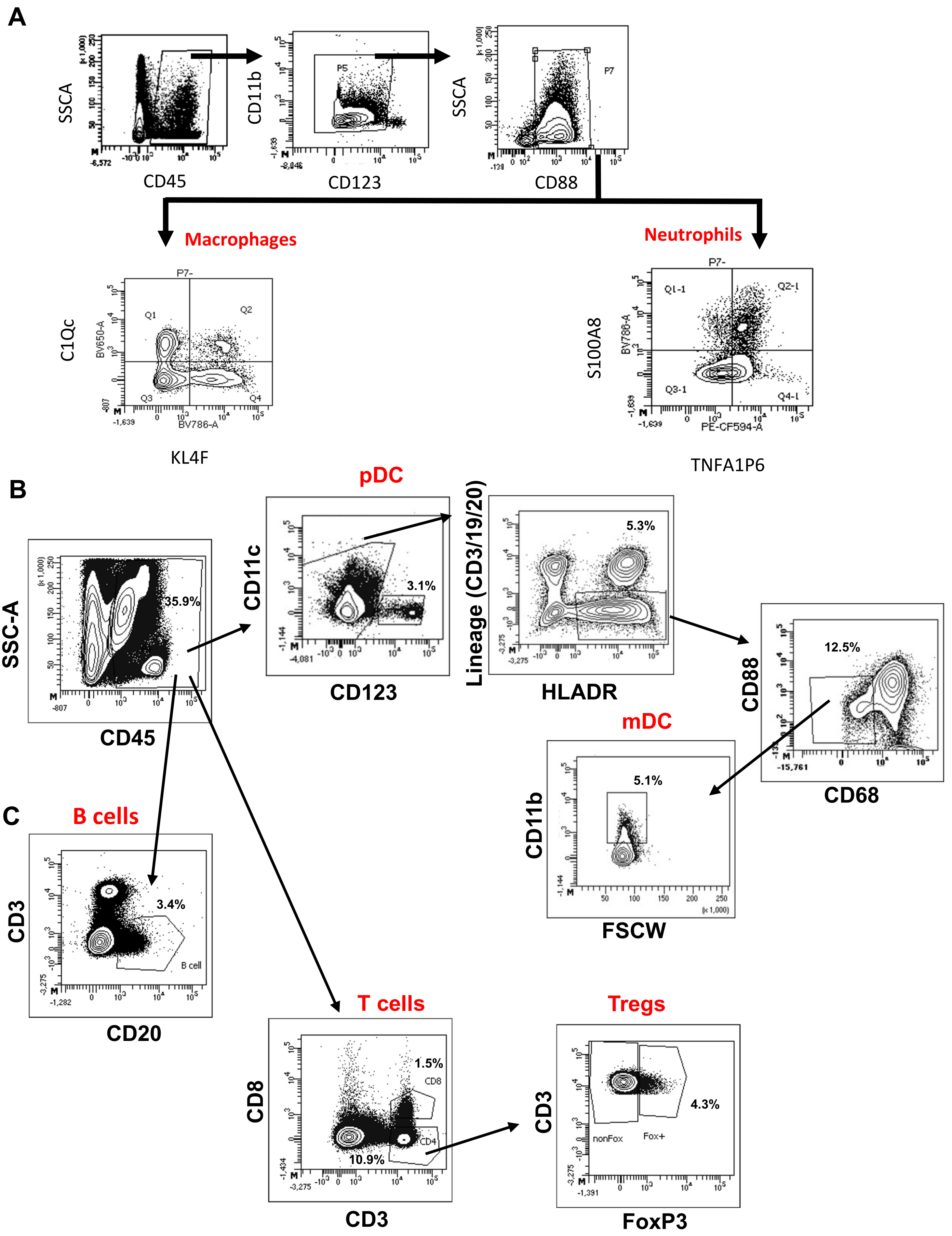

Fig. S2. Flow cytometry gating strategies to identify immune cell populations in the fetal lung. Representative flow cytometry analysis of lung single cell suspensions from an IA LPS animal to identify **(A)** macrophages and neutrophils: macrophages (Interstitial: $CD45^{+}CD123^{-}CD88^{+}C1QC^{+}KLF4^{-}$, Alveolar macrophage: $CD45^{+}CD123^{-}CD88^{+}C1QC^{-}KLF4^{+}$, Inflammatory macrophage: $CD45^{+}CD123^{-}CD88^{+}C1QC^{+}KLF4^{+}$); Neutrophils ($CD45^{+}CD123^{-}CD88^{+}TNFAIP6^{+}S100A8^{+}$). **(B)** DC populations: pDC ($CD123^{+}CD45^{+}$), mDC ($CD11c^{+}Lineage^{-}HLA\text{-}DR^{+}CD88^{-}CD68^{-}CD11b^{+}$). **(C)** Lymphocyte populations: T cells ($CD3^{+}CD8^{-}$ or $CD8^{+}$), regulatory T cells ($CD3^{+}CD8^{-}FoxP3^{+}$) and B cells ($CD20^{+}$).

Fig. S3

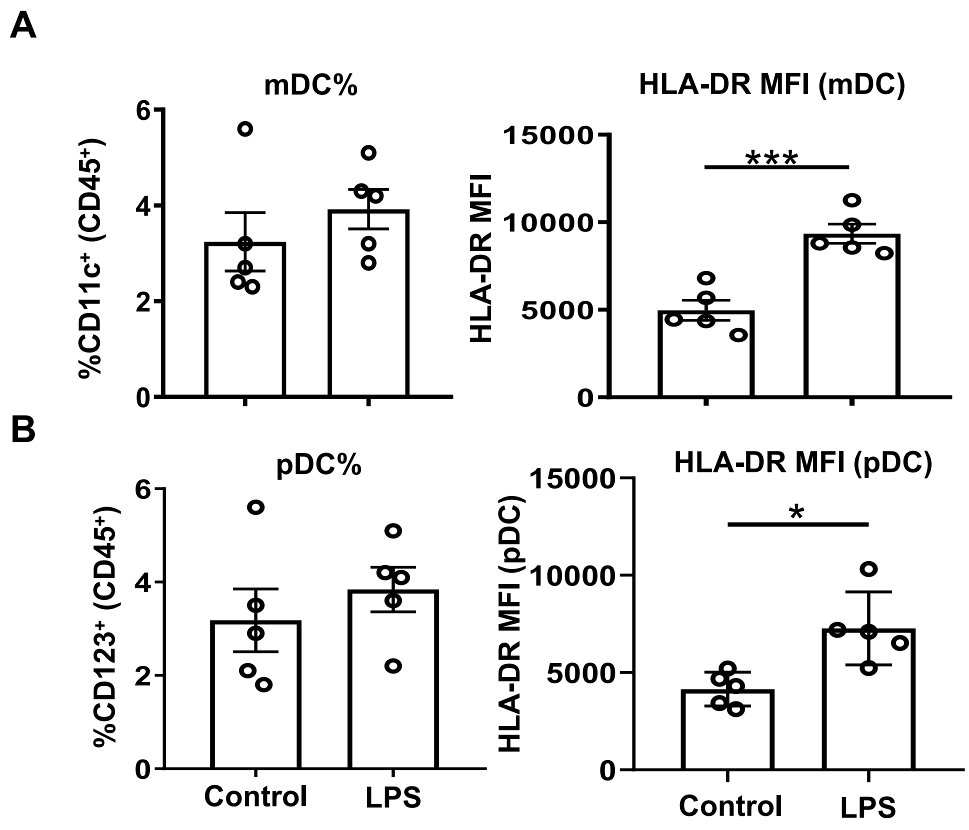

Fig. S3. Dendritic cell response in fetal lung following IA LPS. Percentage and HLA-DR MFI of **(A)** mDC (left and right) and **(B)** pDC (left and right) cells. Each dot represents one animal, with mean (SEM) displayed, Student's unpaired t-test; *p≤0.05, ***p≤0.001.

Fig. S4

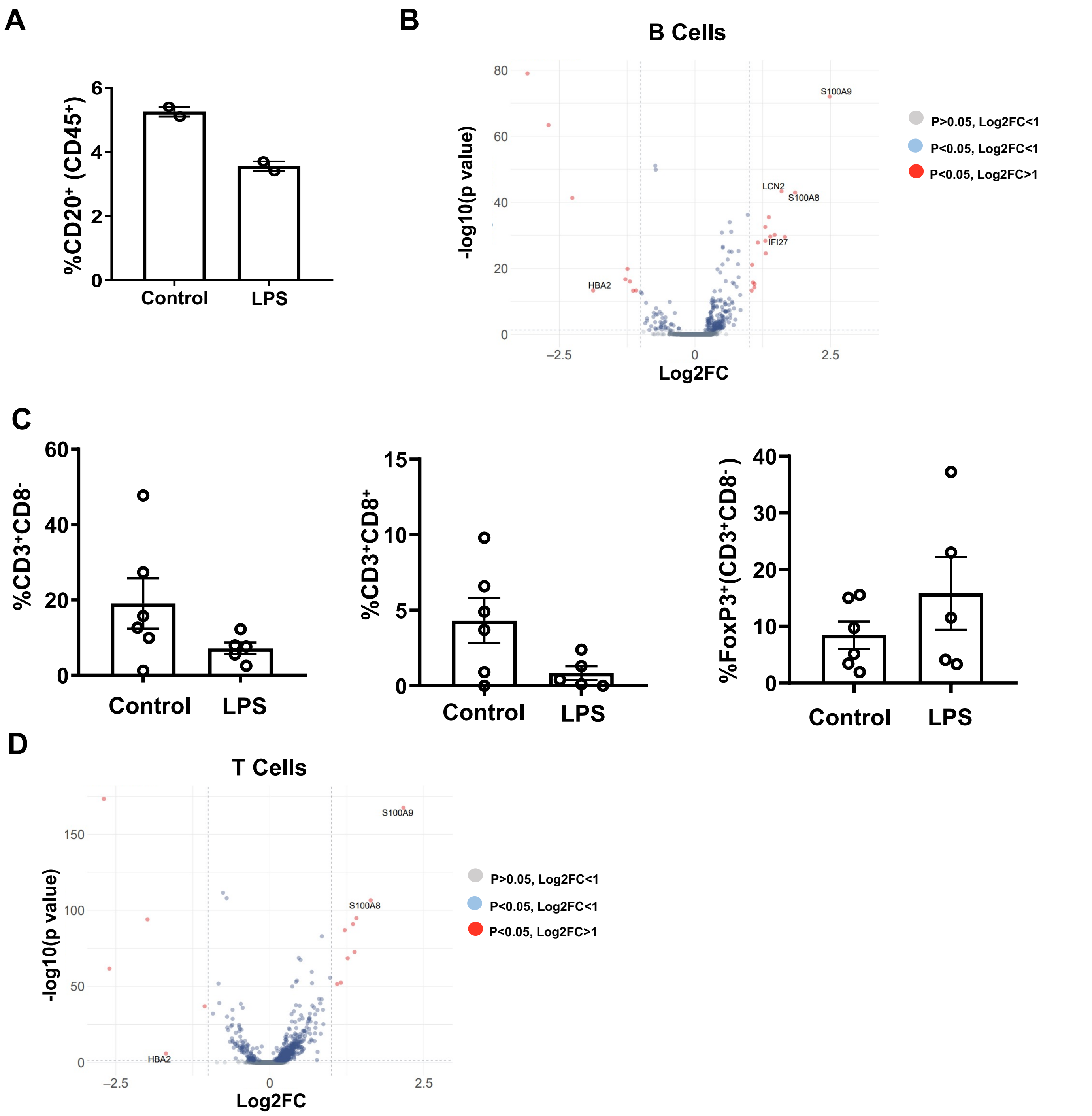

Fig. S4. Lymphoid cell analyses in fetal lung following IA LPS. (A) Percentage and **(B)** volcano plot of DEGs in B cells ($CD20^+$) in control and IA LPS-exposed animals. **(C)** Percentage of $CD4^+(CD3^+CD8^-)$, $CD8^+$, and $FoxP3^+$ T cells and **(D)** volcano plot of total T cell population in control and LPS-exposed animals. Each dot represents one animal, with mean (SEM) displayed .

Fig. S5

A

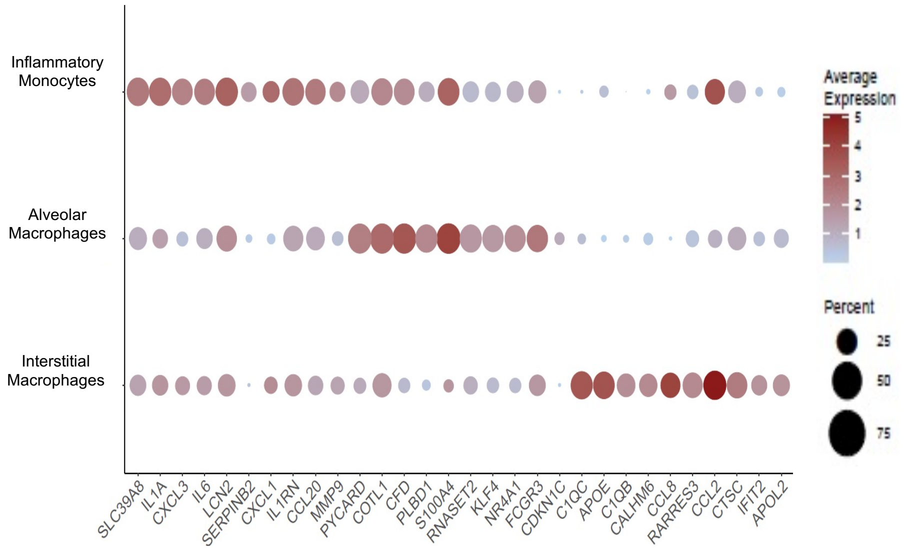

B

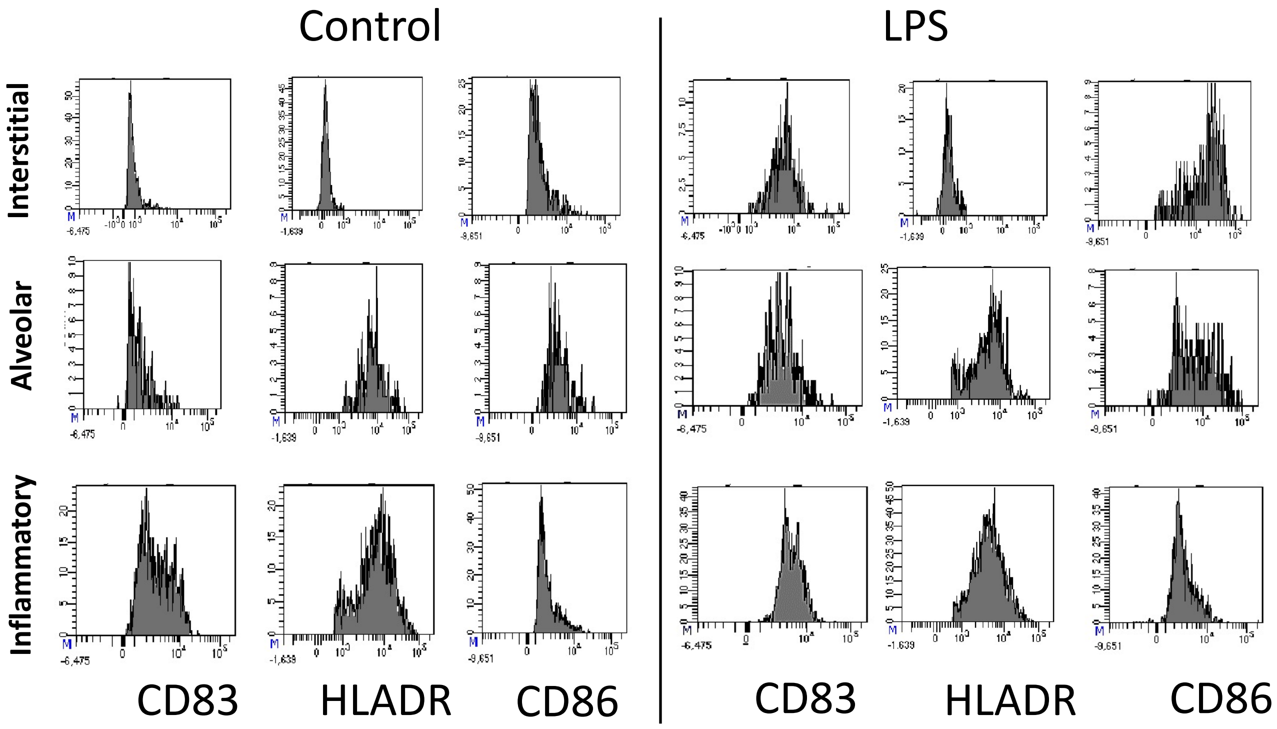

Fig. S5. Characterization of fetal lung monocyte/macrophage populations. (A) Bubble plot of the top 10 conserved genes in the inflammatory monocyte, alveolar macrophage, and interstitial macrophage populations in the fetal lung of IA LPS exposed fetuses. **(B)** representative expression of CD83, HLA-DR, and CD86 expression in the different monocyte/macrophage populations in lungs of a control or IA LPS animal.

Fig. S6

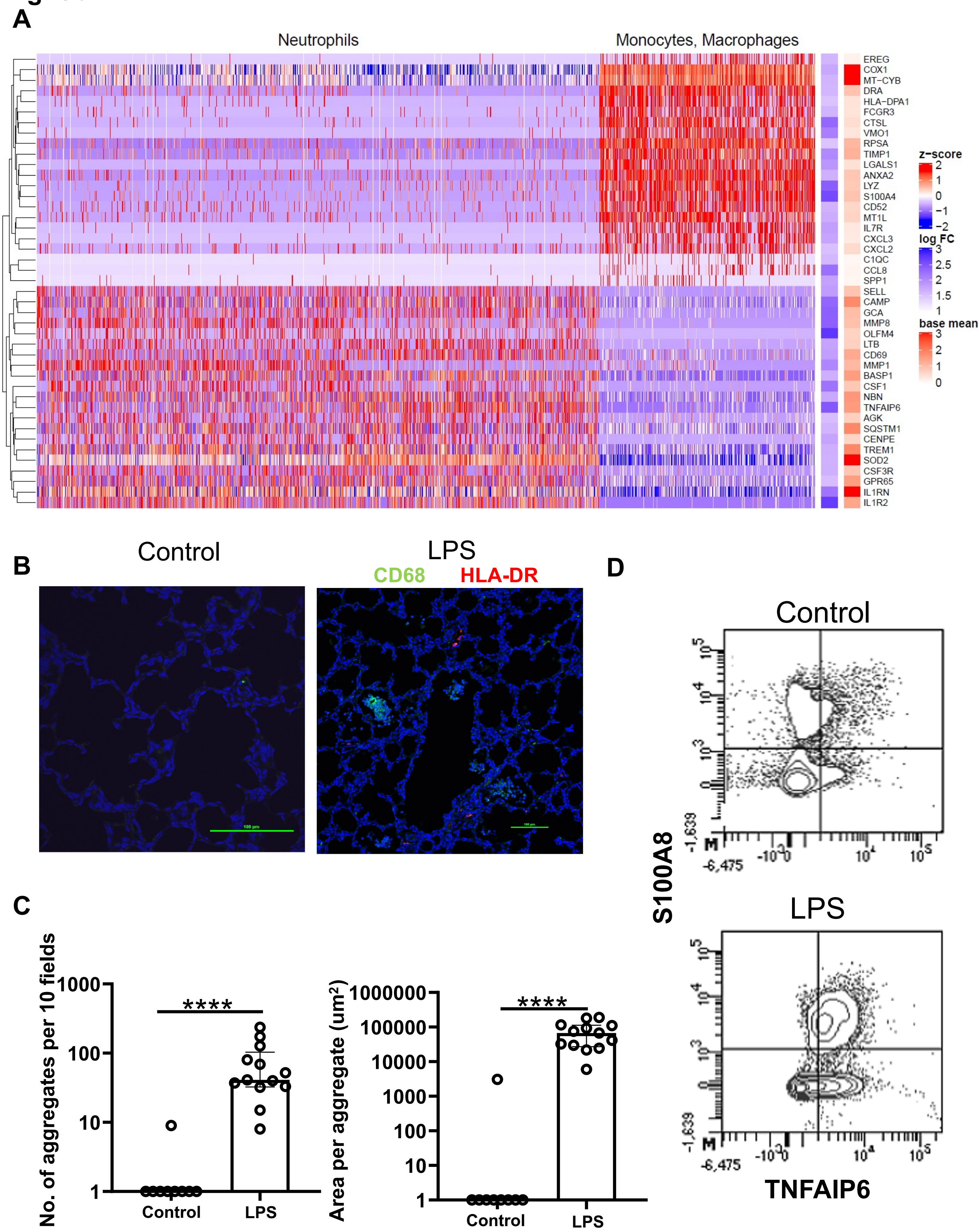

Fig. S6. Characterization of neutrophils in the fetal lung of IA LPS exposed animals. (A) Heat map of top 43 genes (fold change ≥1.5) in neutrophils and monocytes/macrophages in the fetal lung of IA LPS exposed animals. **(B)** Representative images of control and IA LPS fetal lung stained for CD68 and HLA-DR (40X); scale bar is 100μm. **(C)** Neutrophil aggregate count (left) and area (right) in the fetal lungs of control and IA LPS fetuses. Each dot represents one animal, with median and interquartile ranges displayed, Mann-Whitney U test; ****p≤0.0001. **(D)** Representative flow plots of neutrophils in the fetal lung of control (top) and IA LPS (bottom) exposed fetuses based on S100A8 and TNFAIP6 expression.

Fig. S7

A

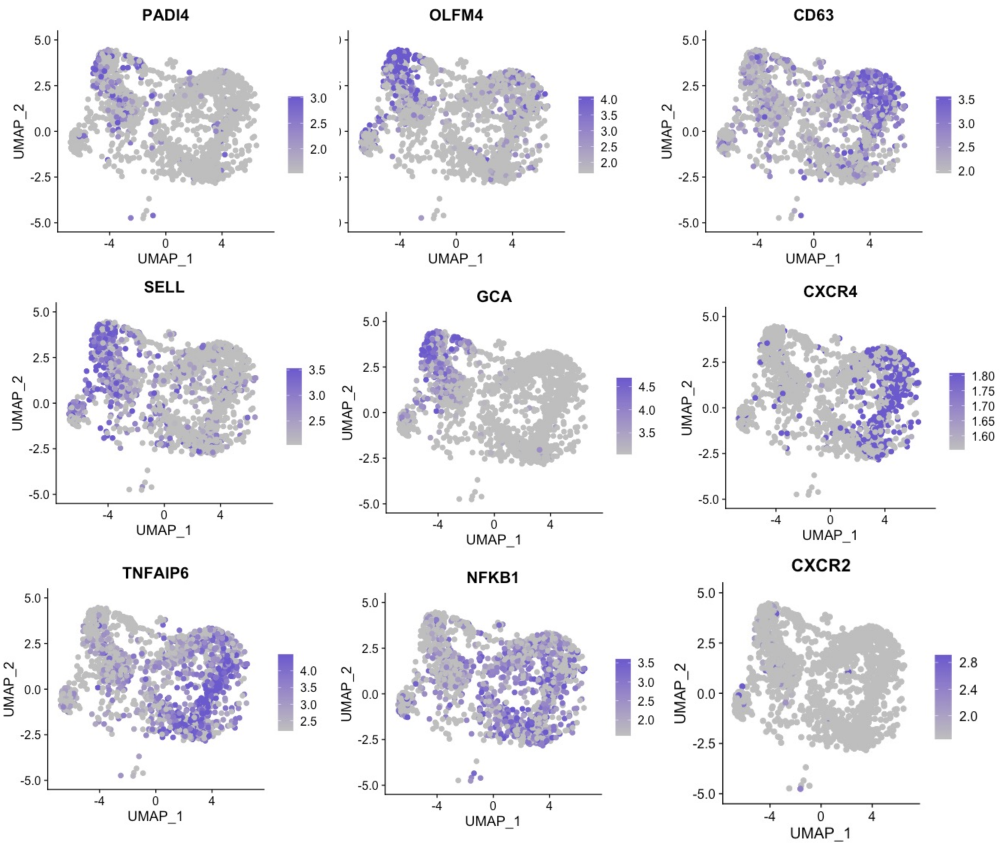

B

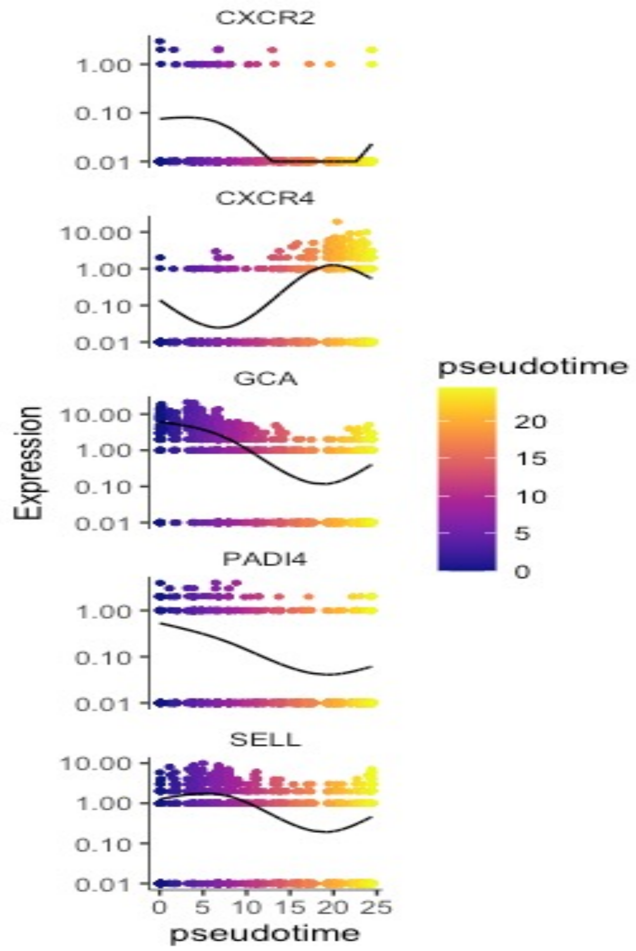

Fig. S7. Characterization of fetal lung neutrophils. (A) Feature plot of gene expression across neutrophil clusters. **(B)** Scatter plots showing expression of selected cluster-defining genes across pseudotime in fetal lung neutrophils following IA LPS exposure.

Fig. S8

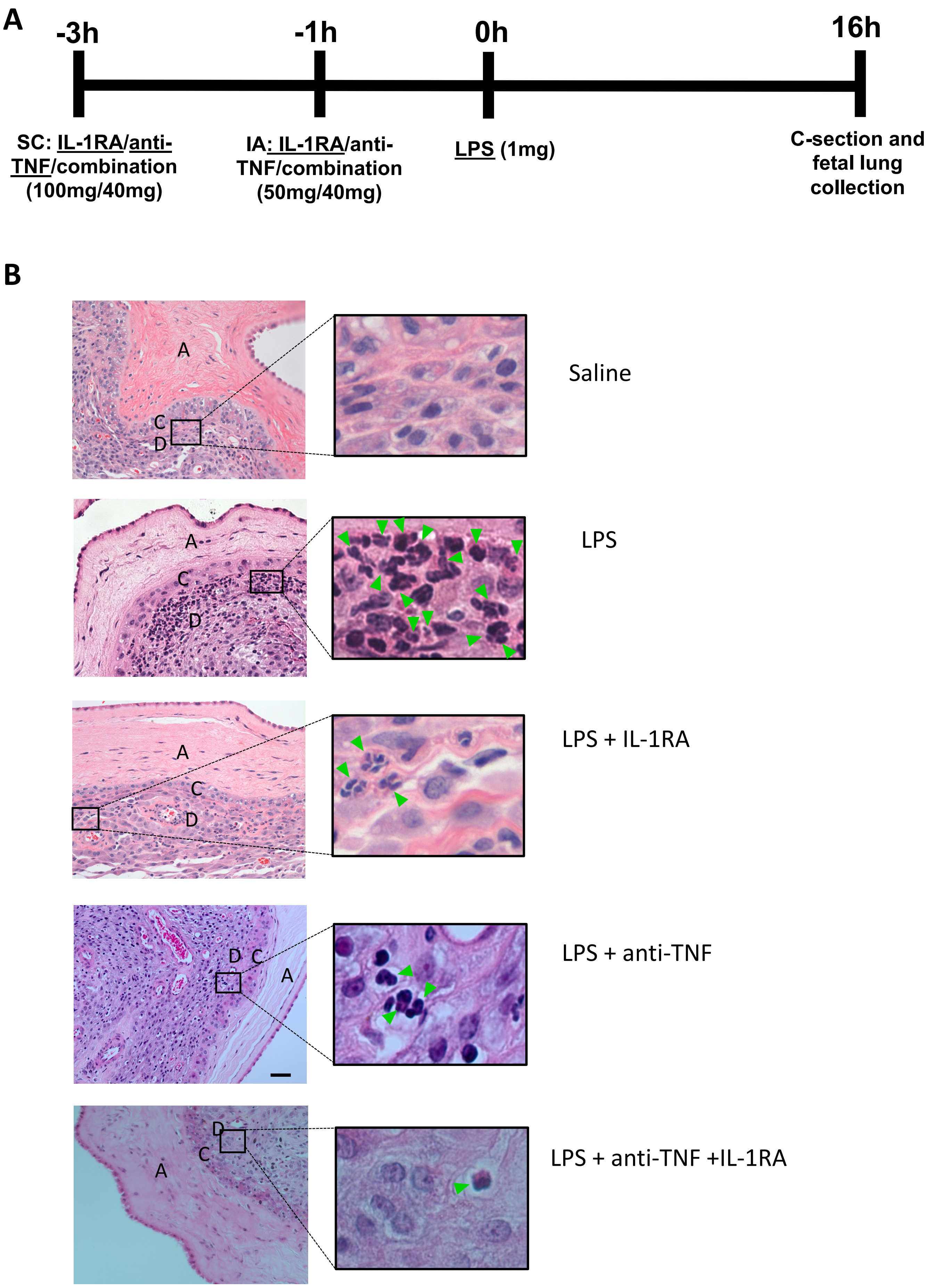

Figure S8. (A) Scheme of treatment administration. **(B) Representative fetal membranes (n=5/each) H&E histology in each condition.** Green arrowheads show neutrophils. A=Amnion; C=Chorion; D=Decidua. Bar = 50 μm

Fig. S9

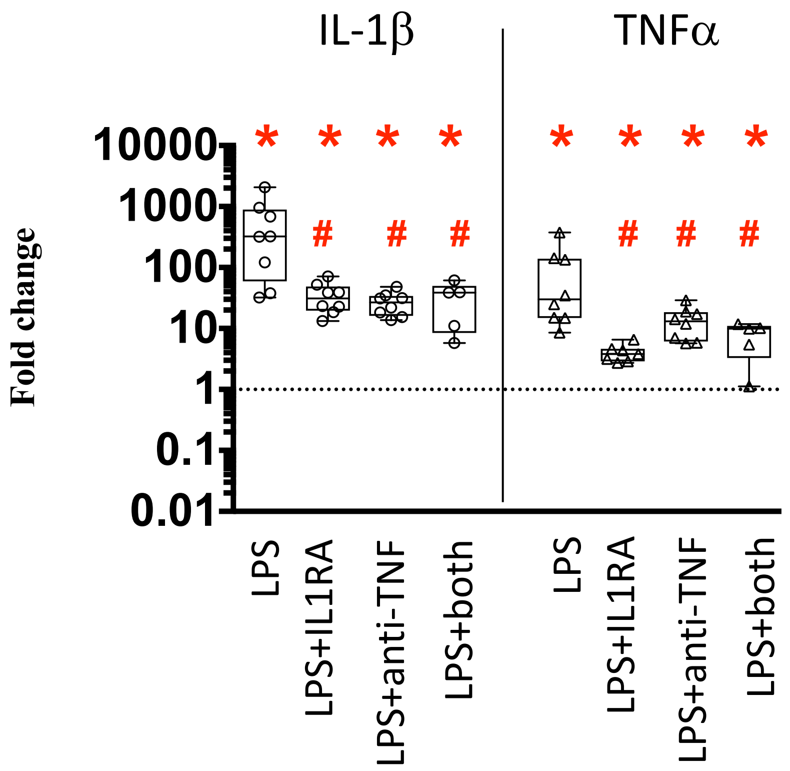

Fig. S9: Blockades (IL-1RA, anti-TNF, alone or combined) blunt mRNA expression of IL-1β and TNFα in the chorioamnion-decidua. qPCR was performed using rhesus-specific Taqman probes. The values were first normalized to the endogenous 18S RNA expression. Box plots show fold change of expression normalized to mean expression in 8 control animals (represented by the dotted line). Each dot represents one animal. *: $p < 0.05$ vs. ctrl; #: $p < 0.05$ vs. LPS (Mann-Whitney U tests).

Fig. S10

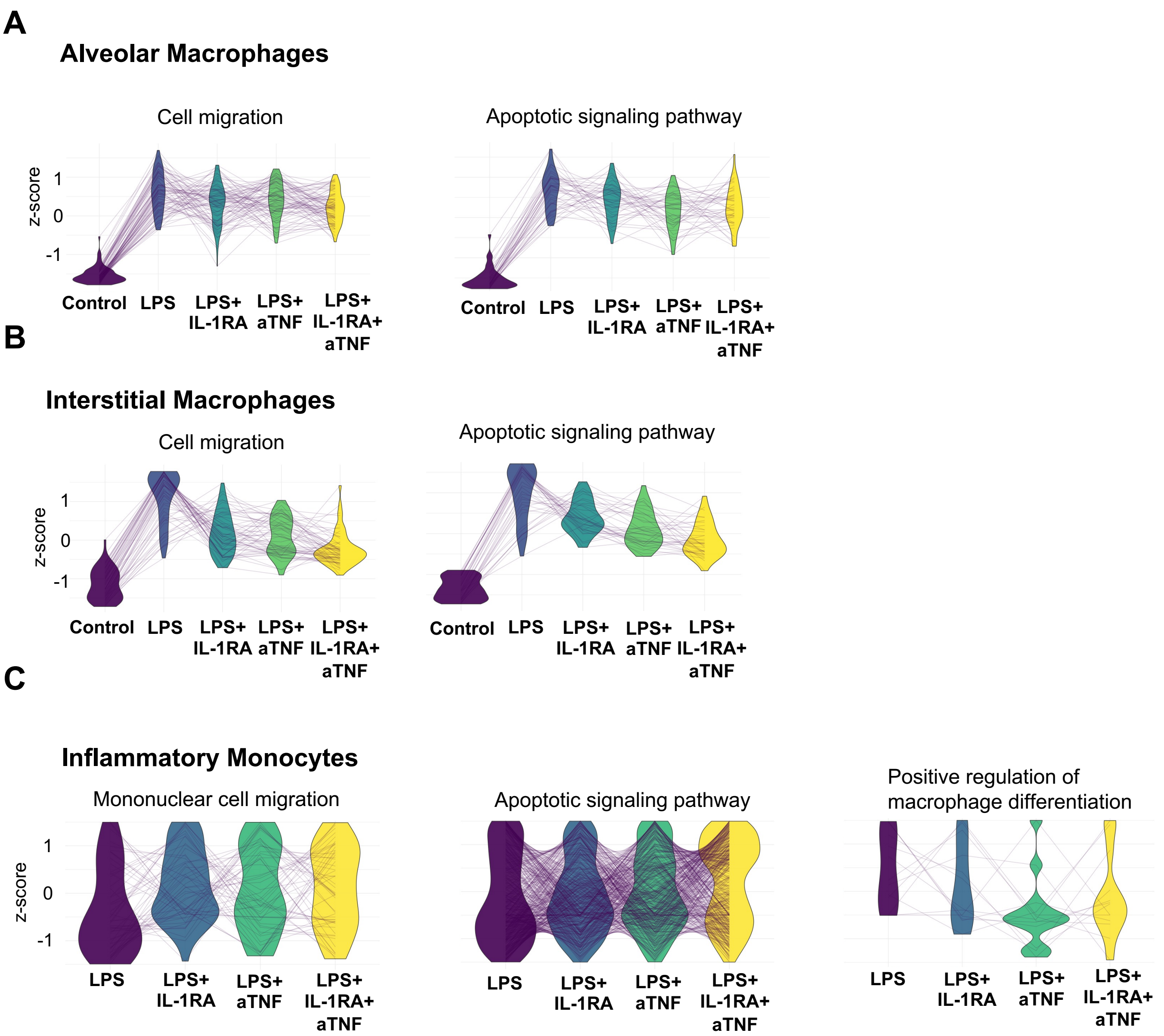

Fig. S10. Transcriptional profile of monocyte/macrophage populations in the fetal lung across treatment conditions. Parallel coordinate plots of scaled expression of representative genes in select biological processes across treatment conditions in the alveolar macrophages (A), interstitial macrophages (B) and inflammatory monocytes (C).

Fig. S11

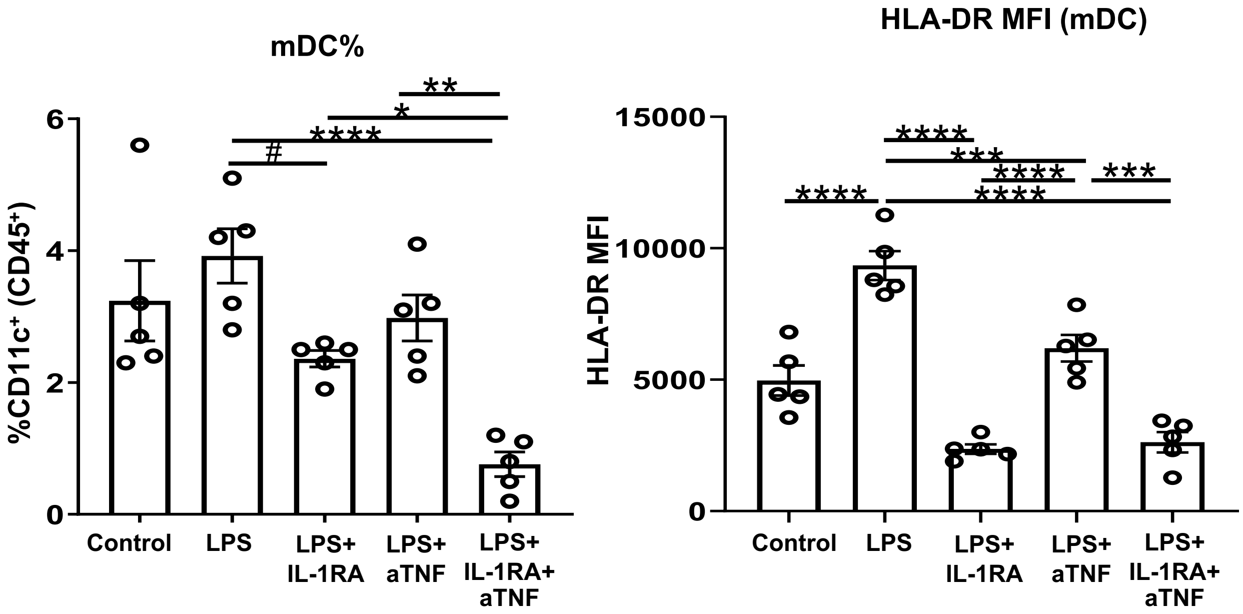

Fig. S11. mDC changes in fetal lung following blocking IL-1 and TNF signaling, alone or in combination. Percentage of mDCs within the $CD45^+$ population across treatment (left) and their expression of HLA-DR MFI (right). Each dot represents one animal, with means (SEM) displayed. Statistical analyses were performed using one-way ANOVA; #p≤0.1; *p≤0.05, **p≤0.01, ***p≤0.001, ****p≤0.0001.

Fig. S12

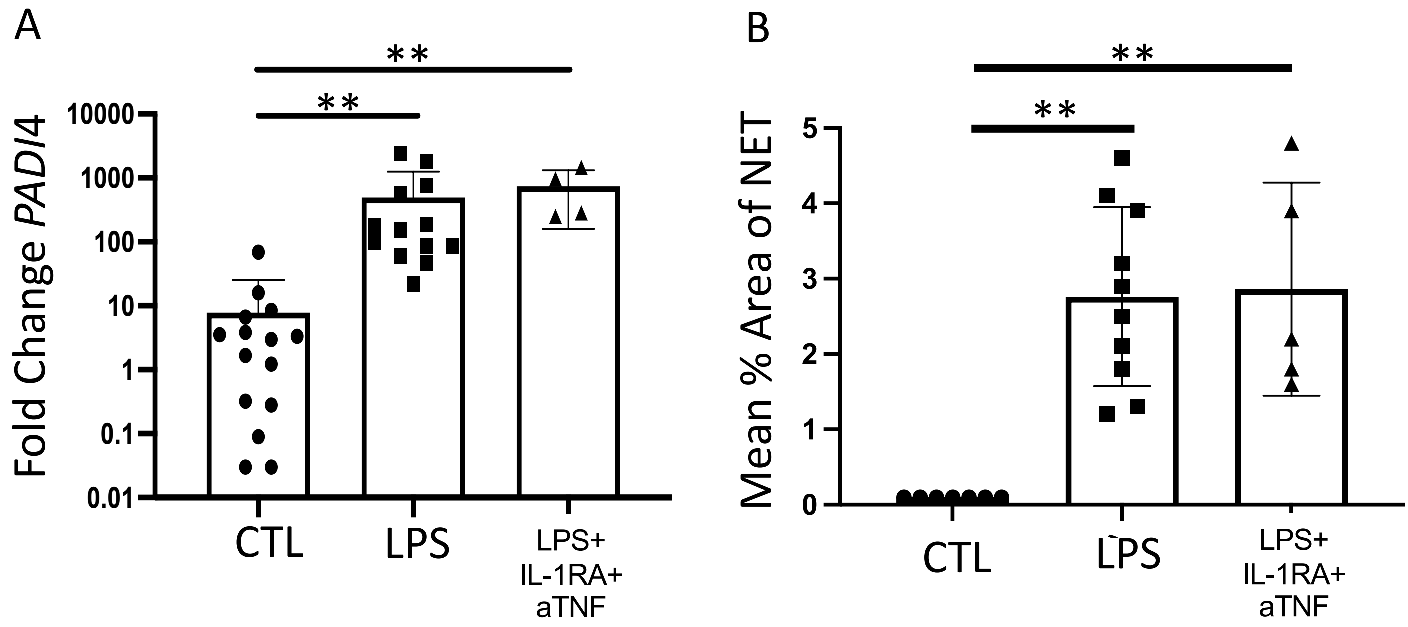

Fig. S12. Combined blockade of IL-1 and TNF signaling did not affect NETosis formation. (A): *PADI4* mRNA expression in the fetal lung of control, LPS, and LPS+IL-1RA+anti-TNF animals. (B): Mean % area of NET in the fetal lungs of control, LPS, and LPS+IL-1RA+anti-TNF animals. Each symbol represents one animal, with means (SEM) displayed. Statistical analyses were performed using one-way ANOVA; **p≤0.01.

Table S1. Demographic data of fetal animal used in the study

	*Controls (n=24)	LPS (n=21)	LPS+IL-1RA (n=10)	LPS+aTNF (n=14)	LPS+IL-1RA+ aTNF (n=5)
Fetal gestational age (days)	132±0.5	132±0.6	132±0.9	131±1.0	133±0.8
Fetal birth weight (g)	330±5.9	330±9.5	338±13.3	355±19.4	359±17.3
Fetal sex (%female)	33	67	60	43	20

mean±SEM; *One control animal is missing gestational age

Table S2. Cytokine production in alveolar wash.

ng/mL	Controls (n=20-24)	LPS (16hr) (n=19-21)	LPS+IL-1RA (n=8-10)	LPS+aTNF (n=13-14)	LPS+IL-1RA+ aTNF (n=4-5)
TNFα*+	0.0038 (0.0001-0.021)	0.448 (0.0316-3.713)	0.825 (0.0311-4.595)	0.052 (0.0036-0.097)	0.039 (0.030-0.059)
IL-6*	0.295 (0.0042-2.677)	3.148 (0.330-17.40)	1.293 (0.114-3.253)	1.422 (0.153-7.365)	1.146 (0.293-2.802)
IL1β*	0.00062 (0.0001-0.001)	0.464 (0.0042-4.546)	0.332 (0.003-0.696)	0.151 (0.0030-1.312)	0.055 (0.016-0.147)
GM-CSF*	0.0017 (0.0004-0.009)	0.539 (0.059-2.540)	0.189 (0.118-0.275)	0.369 (0.0098-2.257)	0.107 (0.021-0.233)
IL-8*	0.0976 (0.0072-0.708)	18.58 (1.963-110.4)	14.50 (2.309-15.72)	5.642 (0.4417-12.53)	4.281 (1.604-9.286)
CCL2*	0.173 (0.0402-0.507)	20.48 (0.0073-32.83)	5.069 (1.248-10.31)	8.832 (0.854-48.90)	4.665 (1.357-10.85)
IL-10*	0.0026 (0.0009-0.009)	0.068 (0.0036-0.190)	0.018 (0.0052-0.066)	0.039 (0.001-0.205)	0.062 (0.0094-0.109)

Data presented as mean and range, comparisons made using Kruskai-Wallis test; *control v. lps $p \le 0.0001$, + lps v. lps+aTNF $p \le 0.05$

Table S3. Cytokine mRNA expression in the fetal lung..

mRNA relative expression	Controls (n=18)	LPS (16hr) (n=15)	LPS+IL-1RA (n=6-9)	LPS+aTNF (n=6-9)	LPS+IL-1RA+ aTNF (n=5)
TNFα*	1 (0.30-4.5)	179 (24.1-421.2)	186 (14.4-369.1)	80 (9.9-153.6)	165 (16.4-581.5)
IL-6*	1 (0.53-2.2)	1381 (128.2-2977.8)	621 (60.2-963.5)	458(18.6-1311.9)	157 (26.2)
IL1β*	1 (0.57-1.8)	1021 (191.1-3039.4)	984 (45.9-1403.3)	423(48.2-790.9)	420 (79.0-1256.1)
IL-8*	1 (0.33-2.7)	2993 (329.8-8800.6)	3377 (159.3-8663.4)	827 (58.1-2120.9)	753 (85.4-2815.9)
CCL2*	1 (0.02-2.5)	138 (11.6-606.0)	90 (9.7-191.5)	43 (3.1-142-9)	91 (15.9-284.4)

Data presented mean and range, comparisons made using Kruskai-Wallis test; *control v. lps $p \leq 0.0001$

Table S4. Demographic data of fetal animals used for scRNAseq

	Controls (n=2)	LPS (n=2)	LPS+IL-1RA (n=3)	LPS+aTNF (n=3)	LPS+IL-1RA+ aTNF (n=3)
*Fetal gestational age (days)	129.5±0.5	130.5±0.5	131.7±0.7	134.00±0	133.0±1.2
*Fetal birth weight (g)	348±48.0	298.4±38.7	301.7±26.1	416.4±32.7	343.1±12.5
Fetal sex (%female)	50	100	66.6	33.3	33.3

* Values are in mean±SEM

Table S5. Antibodies used in flow cytometry experiments

Marker	Clone	Manufacturer
HLA-DR	L243	Biolegend
CD11c	3.9	Biolegend
CD86	IT2.2	Biolegend
CD11b	3.9	Biolegend
CD3	GHI/61	BD Bioscience
CD3	SP34-2	BD Bioscience
CD19	SJ25C1	BD Bioscience
CD20	2H7	BD Bioscience
CD123	7G3	BD Bioscience
CD45	DO58-1283	BD Bioscience
CD8α	RPA-T8	eBioscience
FoxP3	PCH101	eBioscience
LiveDead Aqua	-	eBioscience
CD88	P12/1	Bio-Rad
TNFAIP6	Rabbit Polyclonal	Thermo Fisher Scientific
S100A8	MA5-17623	Thermo Fisher Scientific
C1Q	MA1-40313	Thermo Fisher Scientific
KLF4	PA5-23184	Thermo Fisher Scientific
CD83	HB15e	BD Bioscience
CD68	KP1	Santa cruz
